# Spatiotemporal monitoring of a periodontal multispecies biofilm model: demonstration of prebiotic treatment responses

**DOI:** 10.1128/aem.01081-23

**Published:** 2023-09-28

**Authors:** Justien Ghesquière, Kenneth Simoens, Erin Koos, Nico Boon, Wim Teughels, Kristel Bernaerts

**Affiliations:** 1 Chemical and Biochemical Reactor Engineering and Safety (CREaS), Department of Chemical Engineering, University of Leuven (KU Leuven), Leuven, Belgium; 2 Soft Matter, Rheology and Technology, Department of Chemical Engineering, University of Leuven (KU Leuven), Leuven, Belgium; 3 Center for Microbial Ecology and Technology (CMET), Ghent University (UGent), Gent, Belgium; 4 Department of Oral Health Sciences, University of Leuven (KU Leuven) and Dentistry (Periodontology), University Hospitals Leuven, Leuven, Belgium; University of Illinois Urbana-Champaign, Urbana, Illinois, USA

**Keywords:** oral biofilm, periodontitis, drip flow biofilm reactor, fluorescent proteins, spatiotemporal characterization, prebiotics, L-arginine

## Abstract

Biofilms are complex polymicrobial communities which are often associated with human infections such as the oral disease periodontitis. Studying these complex communities under controlled conditions requires *in vitro* biofilm model systems that mimic the natural environment as close as possible. This study established a multispecies periodontal model in the drip flow biofilm reactor in order to mimic the continuous flow of nutrients at the air-liquid interface in the oral cavity. The design is engineered to enable real-time characterization. A community of five bacteria, *Streptococcus gordonii*-GFPmut3*, *Streptococcus oralis*-GFPmut3*, *Streptococcus sanguinis*-pVMCherry, *Fusobacterium nucleatum*, and *Porphyromonas gingivalis*-SNAP26 is visualized using two distinct fluorescent proteins and the SNAP-tag. The biofilm in the reactor develops into a heterogeneous, spatially uniform, dense, and metabolically active biofilm with relative cell abundances similar to those in a healthy individual. Metabolic activity, structural features, and bacterial composition of the biofilm remain stable from 3 to 6 days. As a proof of concept for our periodontal model, the 3 days developed biofilm is exposed to a prebiotic treatment with L-arginine. Multifaceted effects of L-arginine on the oral biofilm were validated by this model setup. L-arginine showed to inhibit growth and incorporation of the pathogenic species and to reduce biofilm thickness and volume. Additionally, L-arginine is metabolized by *Streptococcus gordonii*-GFPmut3* and *Streptococcus sanguinis*-pVMCherry, producing high levels of ornithine and ammonium in the biofilm. In conclusion, our drip flow reactor setup is promising in studying spatiotemporal behavior of a multispecies periodontal community.

**Importance:**

Periodontitis is a multifactorial chronic inflammatory disease in the oral cavity associated with the accumulation of microorganisms in a biofilm. Not the presence of the biofilm as such, but changes in the microbiota (i.e., dysbiosis) drive the development of periodontitis, resulting in the destruction of tooth-supporting tissues. In this respect, novel treatment approaches focus on maintaining the health-associated homeostasis of the resident oral microbiota. To get insight in dynamic biofilm responses, our research presents the establishment of a periodontal biofilm model including *Streptococcus gordonii*, *Streptococcus oralis*, *Streptococcus sanguinis*, *Fusobacterium nucleatum*, and *Porphyromonas gingivalis*. The added value of the model setup is the combination of simulating continuously changing natural mouth conditions with spatiotemporal biofilm profiling using non-destructive characterization tools. These applications are limited for periodontal biofilm research and would contribute in understanding treatment mechanisms, short- or long-term exposure effects, the adaptation potential of the biofilm and thus treatment strategies.

## INTRODUCTION

Periodontitis is a multifactorial chronic inflammatory disease that can irreversibly damage the tooth-supporting tissues in the oral cavity (1). Affecting up to 50% of the worldwide population, the global burden of periodontitis is high (2). The current hypothesis of periodontal pathogenesis—called polymicrobial synergy and dysbiosis—proposes that a dysbiotic microbial community synergizes to deregulate the host immune response, leading to chronic inflammation and consequently periodontitis (3). Periodontal health and host-community homeostasis are typically associated with a high abundance of Gram-positive facultative anaerobic bacteria, dominated by *Streptococcus* species, which are early colonizers of surfaces in the oral cavity. Their intrinsic adhesins and receptors enable the co-colonization with secondary and late colonizers, such as *Fusobacterium nucleatum* and *Porphyromonas gingivalis*. These are anaerobic bacteria typically considered as being more pathogenic, although they are also present in healthy biofilms (4–6). As such, the polymicrobial synergy and dysbiosis model states that not only the presence of a keystone pathogen (e.g., *P. gingivalis*) but also the complex interplay between bacterial interactions in the community, the environment, and the host triggers the onset of periodontitis, inducing a state of microbial imbalance (3). Revealing the largely unknown mechanisms behind the initiation of dysbiosis and disease development requires well-established *in vitro* model systems which allow for reproducibility by applying controlled environmental conditions. In order to mimic mouth conditions and capture temporal behavior, such systems would benefit from the addition of flow, growth of multispecies biofilms, and non-destructive characterization techniques.

Most multispecies periodontal *in vitro* models are based on statically grown biofilms on glass- or hydroxyapatite-coated substrates (7–9). Being relatively inexpensive and technically simple to set up, static models allow for high-throughput screening and susceptibility applications (10). Notwithstanding their relevance in periodontal research, static models do not consider the continuous flow of saliva to which natural oral biofilms are exposed and the accompanying supply and removal of medium components. To cope with the constantly changing environment in the oral cavity, continuous flow systems such as the constant-depth film fermenter and the artificial mouth model are being applied (11). By continuously trickling medium over the biofilm in a thin film layer, these models have been used to grow *in vitro* multispecies oral biofilms in an environment that mimics the oral cavity (12–15). Closely related to these models, the drip flow biofilm reactor, proposed by Goeres et al. (16), is used to grow biofilms under low shear conditions at the air-liquid interface. Although highly promising, continuous drip flow model applications usually focus on caries research and are limited for multispecies oral biofilms associated to periodontitis (17).

Confocal laser scanning microscopy (CLSM) is generally the preferred technique for capturing biofilm structure and spatial heterogeneities. Combined with fluorescent *in situ* hybridization (FISH), Valm et al. (18) were able to simultaneously visualize 15 different bacterial taxa within human dental plaque. Although FISH can discriminate between multiple active strains within a biofilm, it lacks the possibility of real-time, non-invasive imaging. As an alternative, bacterial cells can be genetically labeled with suitable and specific fluorophores. For streptococcal strains, several optimized and interchangeable fluorescent protein delivery systems proved their value in biofilm experiments, making them highly promising for discriminating between multiple species (19, 20). The widely applied use of fluorescent proteins in periodontal models is hampered, however, by the complex environmental conditions, not always providing the required oxygen for proper maturation of the fluorescent protein (21). While most periodontal models use anaerobic conditions (7, 22–25), the surface of an oral biofilm is exposed to aerobic conditions. In Verspecht et al. (26), strict anaerobic species are present in oral biofilms cultured in micro-aerophilic conditions (6% O_2_). This residence is due to the presence of anaerobic micro-environments in which anaerobic species can survive. The use of the green fluorescent protein GFPmut3* in anaerobically grown *Streptococcus gordonii* biofilms of up to 50 µm thickness was assessed by Hansen et al. (27). Alternatively, to cope with the absence of oxygen, Nicolle et al. (22) developed fluorescently labeled *P. gingivalis*, expressing the SNAP-tag which will specifically and covalently bind to suitable synthetic fluorophores regardless of the availability of oxygen. Combining this *P. gingivalis*-SNAP26 strain with *S. gordonii*-GFPmut3*, they were able to grow a bispecies anaerobic biofilm expressing strong fluorescence signal.

The increasing emergence against antibiotic resistance drives the establishment of novel treatment approaches which focus on controlling or restoring health-associated homeostasis, such as prebiotics (28). Prebiotics envisage a nutritional stimulation of beneficial bacteria, conferring a health benefit to the host (29). In this context, exogeneous arginine addition has emerged as an effective therapy for dental plaque control (30). Research has primarily focused on the ability of arginolytic micro-organisms to metabolize L-arginine via the arginine deiminase pathway (ADS) producing ammonia (31). As such, arginine metabolism neutralizes the pH and counteracts the deleterious effects of tooth demineralization (32–34). Additionally, for single *S. gordonii* biofilms, exogeneous arginine treatment reduces biofilm biomass (35) and causes earlier biofilm detachment (36). Destabilizing effects were also observed in multispecies biofilms in terms of reduced extracellular polymeric substances (EPS) formation and biofilm volumes, changing the biofilm architecture (37, 38). Interestingly, arginine alters the composition of a saliva-derived biofilm toward higher proportions of *Streptococcus* and *Veillonella* species, while proportions of Gram-negative organisms were reduced (37). Altogether, arginine exerts multifaceted effects on oral biofilms concerning structural, metabolic, as well as compositional changes.

This paper aims to develop and dynamically characterize a five-species periodontal biofilm model which mimics the continuous supply and removal of nutrients in the oral cavity. Five key species are selected, including three beneficial streptococcal strains and two pathogenic species. More specifically, *S. gordonii*, *Streptococcus oralis*, and *Streptococcus sanguinis* are widely studied organisms in periodontal research as they are initial colonizers and abundantly present in periodontal health conditions (4–6). *F. nucleatum*, also present in high numbers in healthy individuals (6), is a mid-colonizer with the ability to bind to several other species and is associated with periodontal disease (39). *P. gingivalis* is a late colonizer and the keystone pathogen for periodontitis (40). This selection ensures a representative community for periodontitis and a manageable model complexity to identify key phenomena and interactions. The drip flow biofilm reactor, inspired by Goeres et al. (16), is engineered in such a way to enable real-time profiling of metabolites, biofilm structure, and cell abundances and numbers. The proof of concept for the model is performed with L-arginine treatment, a proven prebiotic compound for periodontitis. The proposed model with the characterization platform has the advantage of combining long-term biofilm experiments under flow and aerobic conditions, non-invasive characterization techniques, and more than three fluorescently labeled species.

## RESULTS

### Controlled establishment of a biofilm in the drip flow reactor

**Fig 1:**
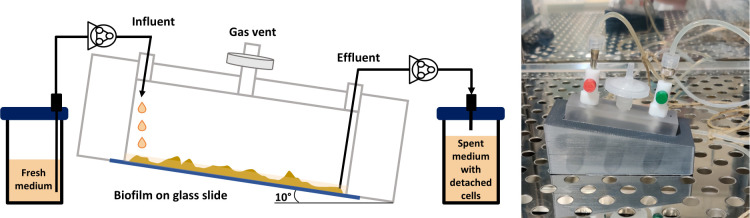
Drip flow biofilm reactor setup.

**Fig 2:**
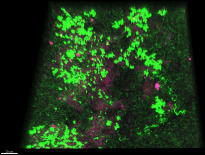
Example of a confocal image used for image analysis. *S. gordonii*-GFPmut3*, *S. oralis*-GFPmut3*, and *F. nucleatum* in green, *S. sanguinis*-pVMCherry in magenta, and *P. gingivalis*-SNAP26 in red. Scale bar is 20 µm.

**Fig 3:**
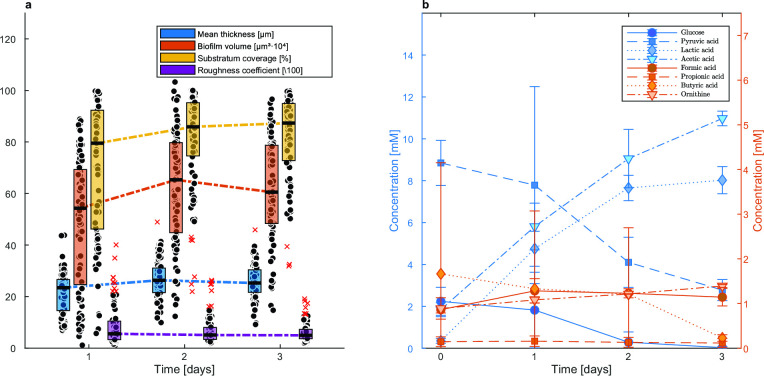
Spatiotemporal characterization of the five-species periodontal biofilm during the 3 days start-up phase of the drip flow biofilm reactor. (**a)** Time profiles of structural biofilm features. Box plots for three biological replicates are shown, including 28 confocal images per replicate per time point. Box plots show the first (lower) quartile, the median, the third (upper) quartile, and outliers by red crosses. (**b) **Metabolite concentrations in the effluent of the drip flow reactor reflecting metabolic activity of the biofilm. Averages and standard deviations of three biological replicates are shown. Measurements for ornithine only include two biological replicates.

After a short attachment period under recycling flow, the biofilm develops over time under a continuous flow of fresh medium in the drip flow biofilm reactor (Fig. 1). Figure 2 shows an example confocal image of the five-species biofilm that is established in the drip flow reactor after start-up. The green color clusters the signals of *S. gordonii*-GFPmut3*, *S. oralis*-GFPmut3*, and *F. nucleatum*, magenta shows *S. sanguinis*-pVMCherry, and red corresponds to *P. gingivalis*-SNAP26. *F. nucleatum* is present in the green cluster due to its green autofluorescence. Fluorescence compatibility and autofluorescence of *F. nucleatum* are described in Supplementary file S1. Structural features are daily quantified on 28 location-specific confocal images, shown as box plots in Fig. 3a. The spread of measurements on each time point demonstrates the spatial heterogeneous nature of the biofilm. Biofilm mean thickness, volume, and substratum coverage increase over a period of 3 days of biofilm growth to average values of 26 µm, 62·10^4^ µm^3^, and 82%, respectively. The roughness coefficient (a measure for the heterogeneity across the biofilm) slightly decreases and reaches a value of 0.06. Stable average biofilm features are established, and the decreasing roughness coefficient and decreasing data spread correspond to the development of a more spatially uniform biofilm.

Metabolic activity of the biofilm is reflected in metabolite changes in the medium. After 3 days, all glucose added through the fresh feed medium—the main growth-limiting substrate of the streptococci—is consumed by the biofilm (returning a non-detectable concentration in the effluent, see Fig. 3b). About 6 mM pyruvic acid is consumed from the feed medium. Lactic acid and acetic acid are the main metabolic products reaching concentrations of 8.02 and 10.97 mM, respectively, in the effluent. Other organic acids (formic, propionic, and butyric acid), and ornithine do not accumulate in the effluent. Free and bound amino acids such as alanine, lysine, threonine, and serine are slightly consumed (Supplementary file S2, Fig. A4). At day 3, the standard deviation on each time point, i.e., the average of three biological replicates, becomes small, which means that the setup renders growth of a periodontal biofilm with reproducible metabolic activity. In view of understanding metabolite changes, a growth and metabolic characterization of single species cultures is performed, and a metabolic interaction network is derived (see Supplementary file S3). Glucose is converted by all strains except by *P. gingivalis*-SNAP26. Pyruvic acid is consistently consumed by all strains. Amino acid and peptide consumption and production are observed for *F. nucleatum* and *P. gingivalis*-SNAP26. Acetic and lactic acid are overall the main organic acids, whereas *P. gingivalis*-SNAP26 and *F. nucleatum* are the only butyrate producers. Hydrogen peroxide is produced in significant amounts by all streptococci, and mostly by *S. oralis* (Supplementary file S3, Fig. A7c). Hydrogen peroxide production in the biofilm is also detected since the addition of the Amplex Red solution to the biofilm returns a pink fluorescence signal across the whole biofilm (Supplementary file S2, Fig. A5). Localized detection of H_2_O_2_ is not possible.

**Fig 4:**
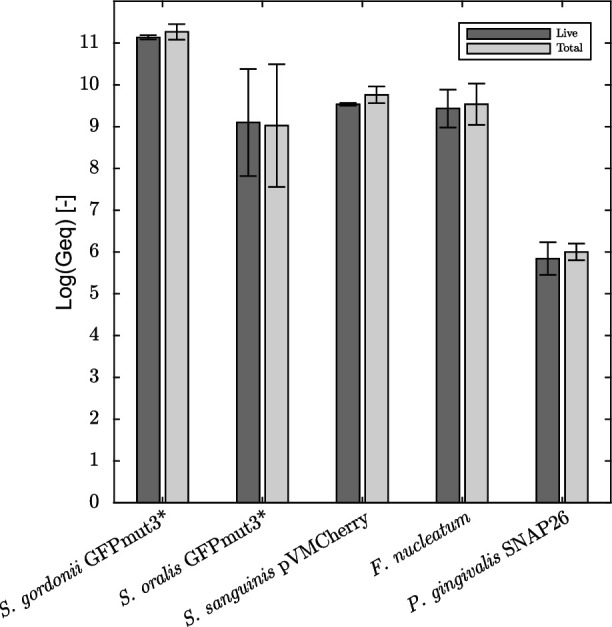
Viable and total cell numbers per species in the biofilm after 3 days. Averages and standard deviations of three biological replicates are shown.

The bacterial biofilm composition is determined at the end of the experiment by extracting the entire biofilm and performing quantitative polymerase chain reaction (qPCR) measurements on it. Figure 4 compares the live and total (i.e., sum of live and dead) numbers of each species. The biofilm community is fully viable (minor differences between live and total cell numbers), and the overall composition is reproducible among biological replicates (small error bars). The biofilm is dominated by the presence of streptococcal strains, while the pathogenic species occupy a smaller fraction. *S. gordonii*-GFPmut3* is present in the highest number, with a live log value of 11.14 genome equivalents, followed by *S. sanguinis*-pVMCherry, with a live log value of 9.53 genome equivalents. Numbers of *S. oralis*-GFPmut3* are a bit more varying between replicate experiments, with an average of 9.10 live log genome equivalents. *F. nucleatum*, being more pathogenic, is also present in high numbers equal to 9.43 live log genome equivalents. The keystone pathogen for periodontitis, *P. gingivalis*-SNAP26 is least present in the biofilm, with a value of 5.84 live log genome equivalents, differing up to 5 log genome equivalents with the dominant streptococcal strains.

### Dynamic responses to L-arginine addition

**Fig 5:**
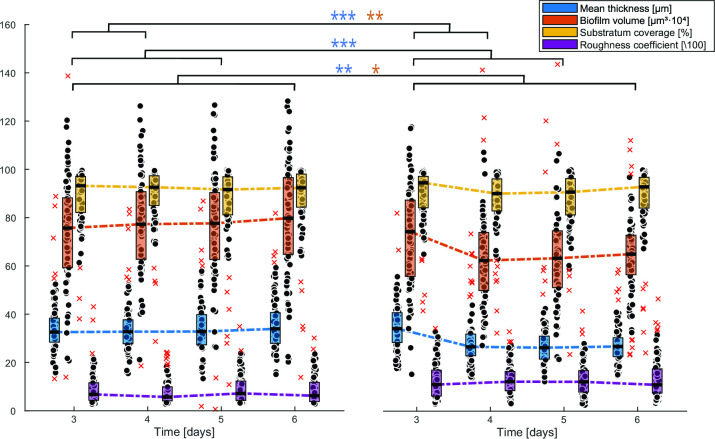
Time profiles of structural biofilm features for the negative control (left) and L-arginine treatment (right). Box plots for three biological replicates are shown, including 28 confocal biofilm images per replicate per time point. Box plots show the first (lower) quartile, the median, the third (upper) quartile, and outliers by red crosses. Statistically significant differences between the negative control and treatment compared to the reference point (day 3) are indicated by *(*P* < 0.05), **(*P* < 0.01), or ***(*P* < 0.001) in the color corresponding to the biofilm feature.

**Fig 6:**
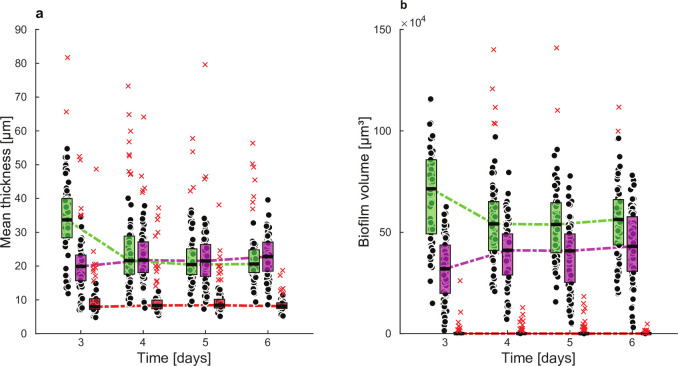
Time profiles of structural biofilm features for the L-arginine treatment experiment per color group of bacteria: (a) mean thickness, and (b) biofilm volume. *S. gordonii*-GFPmut3*, *S. oralis*-GFPmut3*, and *F. nucleatum* in green, *S. sanguinis-*pVMCherry in magenta, and *P. gingivalis*-SNAP26 in red. Box plots for three biological replicates are shown, including 28 confocal biofilm images per replicate per time point. Box plots show the first (lower) quartile, the median, the third (upper) quartile, and outliers by red crosses.

Prolonged operation of the drip flow reactor demonstrates that structural biofilm features remain stable for 3–6 days when the feed composition is not changed. Figure 5 (left) shows constant structural characteristics in terms of mean thickness, biofilm volume, substratum coverage, and roughness coefficient for this negative control (no treatment). On the contrary, a treatment with L-arginine clearly affects the biofilm structure. Starting from a 3-day stable, well-established condition, the biofilm is subjected to 1.5% L-arginine through the medium stream (treatment). This concentration of L-arginine is chosen to be clinically relevant, e.g., in reference (41). Compared to the reference status, the biofilm mean thickness and biofilm volume significantly drop already after 1 day of treatment. The average thickness and biofilm volume reduce with 6.19 µm and 9.26·10^4^ µm^3^, respectively. Furthermore, there is a tendency toward a decrease in substratum coverage and increase in roughness coefficient, corresponding with a more spatially heterogeneous biofilm. Looking at the mean thickness and biofilm volume determined for each color channel (Fig. 6), it becomes clear that the total decreases mainly result from decreases in the green channel, represented by *S. gordonii*-GFPmut3*, *S. oralis*-GFPmut3*, and *F. nucleatum*. The total reduction in biofilm volume is even partly elevated by an increased volume occupation by *S. sanguinis*-pVMCherry. In line with the global structural changes, color effects are most pronounced after 1 day of treatment.

**Fig 7:**
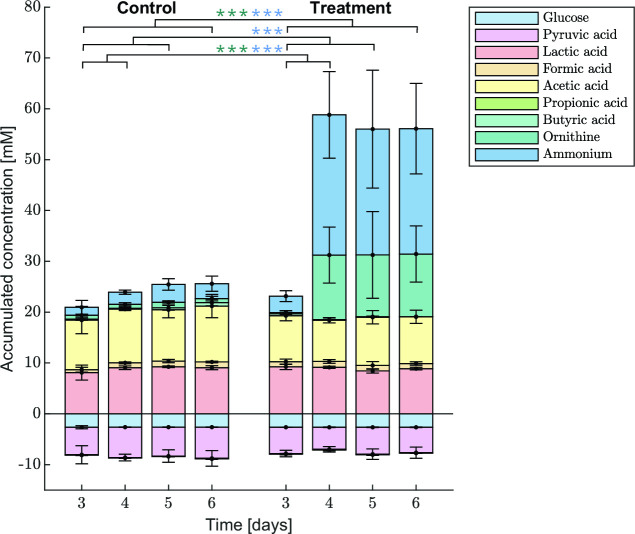
Accumulated metabolite concentrations for the negative control and L-arginine treatment. Metabolites concentration differences between effluent and feed stream represent the metabolic activity of the biofilm. Averages and standard deviations of three biological replicates are shown. Statistically significant differences between the negative control and treatment compared to the reference point (day 3) are indicated by ***(*P* < 0.001) in the color corresponding to the metabolite.

Figure 7 shows the accumulated metabolite concentrations (i.e., the difference between the effluent and feed concentration) which represent the net conversion of metabolites by the whole biofilm community. For the negative control, metabolite consumption and production remain constant over time, implying that the metabolic activity of the biofilm is maintained over 6 days. When the biofilm is exposed to medium with L-arginine, non-significant shifts are observed for glucose and organic acid concentrations, but the ornithine and ammonium production significantly increase. From 86 mM L-arginine in the feed, on average 12 mM and 25 mM are converted to ornithine and ammonium, respectively, as measured in the effluent medium. The alkaline products ornithine and ammonium result from the degradation of citrulline in the arginine deiminase system (42). Measurements correspond to the 1:1 and 1:2 stoichiometry for arginine to ornithine and ammonium conversion, respectively.

**Fig 8:**
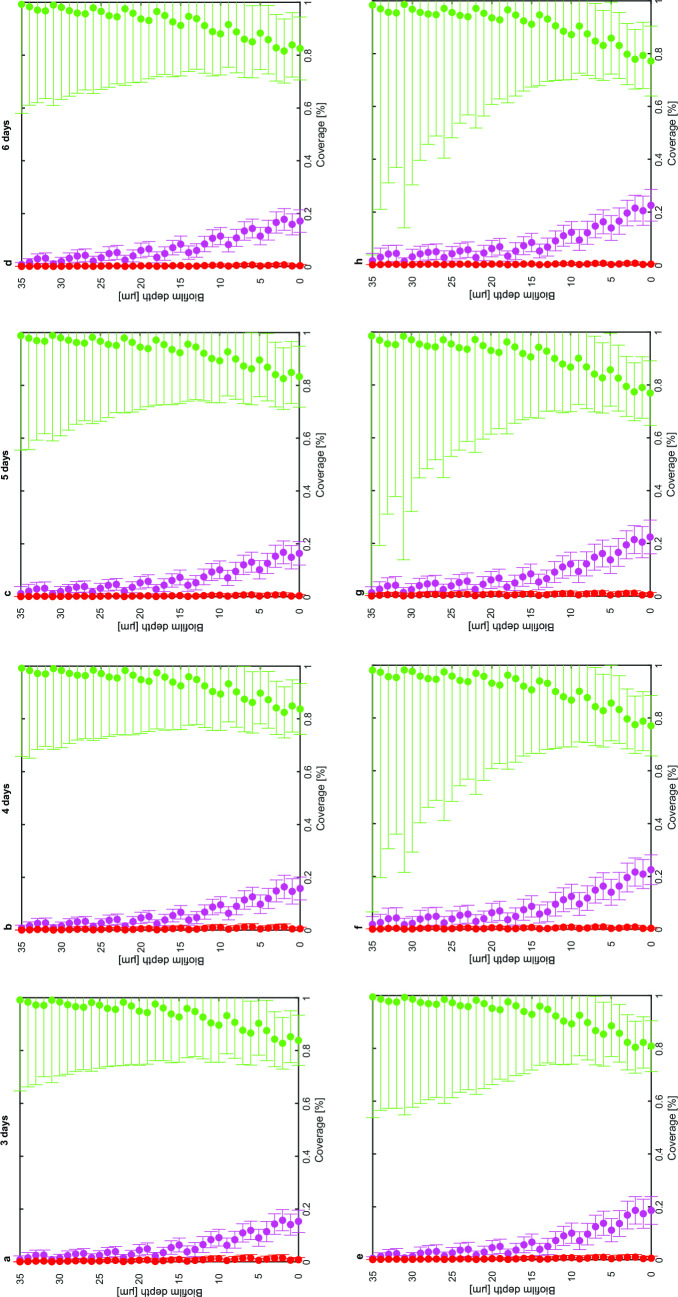
Depth profiles illustrating the color coverages at each height of the biofilm (and thus the spatial composition) over time for the negative control (top row) and L-arginine treatment (bottom row). *S. gordonii-*GFPmut3*, *S. oralis*-GFPmut3*, and *F. nucleatum* in green, *S. sanguinis*-pVMCherry in magenta, and *P. gingivalis*-SNAP26 in red. Time point measurements are performed on 28 confocal images per 3 biological replicates. Statistics are summarized in Supplementary file S4, Table A1.

To get insight into the spatial composition of the biofilm, the relative color percentages over the entire depth of the biofilm are calculated and plotted in Fig. 8. For the negative control, bacterial abundances remain quite constant both in time and across the biofilm depth. On average, 80%–99% of the quantified color pixels is attributed to the green channel, representing *S. gordonii*-GFPmut3*, *S. oralis*-GFPmut3*, and *F. nucleatum*, 1%–20% is accounted to *S. sanguinis*-pVMCherry, and less than 1% is accounted to *P. gingivalis*-SNAP26. All bacteria show a depth dependence across the biofilm depth. *S. sanguinis*-pVMCherry and *P. gingivalis*-SNAP26 are most abundant at the bottom (graphs with a close-up of low coverage percentages are illustrated in Supplementary file S2, Fig. A6). The group of *S. gordonii*-GFPmut3*, *S. oralis*-GFPmut3*, and *F. nucleatum* are abundantly present across the entire biofilm depth and dominate the upper layers. The addition of L-arginine slightly shifts the spatial distribution of the bacteria, but actual shifts are difficult to visualize. However, the standard deviation of the green color increases with (much) higher values at the top of the biofilm and already observed after 1 day of treatment. This increased variation aligns with an increased spatial heterogeneity at the upper part of the biofilm.

**Fig 9:**
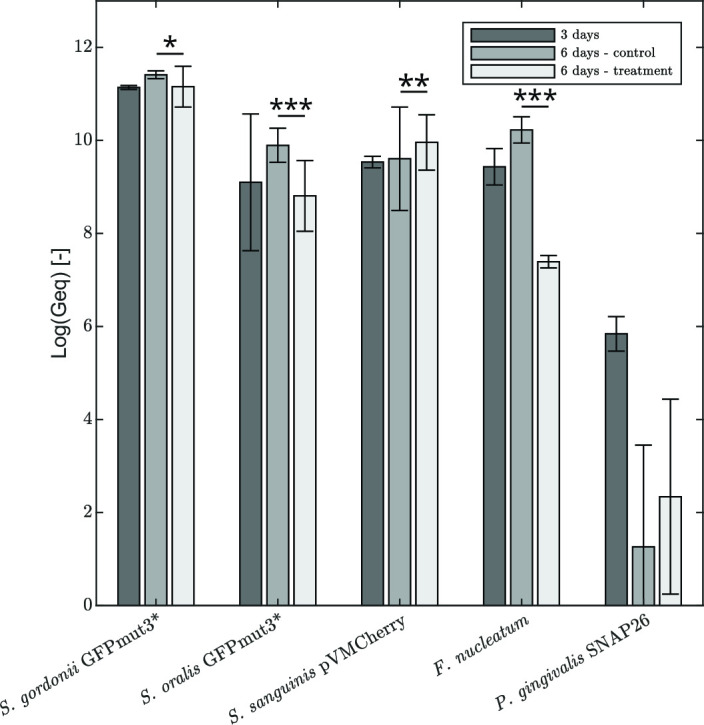
Viable cell numbers per species in the biofilm before treatment (day 3), after L-arginine treatment (day 6) and for the negative control (day 6). Averages and standard deviations of three biological replicates are shown. Statistically significant differences between the negative control and treatment after 6 days are indicated by *(*P* < 0.05), **(*P* < 0.01), or ***(*P* < 0.001).

At day 6, the biofilm of the negative control and the treatment condition are extracted to determine the overall bacterial composition via vitality qPCR (Fig. 9). In the negative control, a slight increase in total cell numbers can be observed for all species, except for *P. gingivalis*-SNAP26 which decreases in cell numbers over the time and becomes variable among the biological replicates. Given the low number, also the qPCR quantification becomes more stochastic. When the biofilm is exposed for 3 days to a continuous treatment with 1.5% L-arginine, all species—except for *S. sanguinis*-pVMCherry—decrease as compared to the negative control at day 6. Most remarkable, *F. nucleatum* is lowered 2.88 live log values. Relative abundances of *S. gordonii*-GFPmut3* and *S. sanguinis*-pVMCherry increase as a result of L-arginine addition (see Supplementary file S3).

## DISCUSSION

This study designed a multispecies periodontal model in the drip flow biofilm reactor combining (i) the simulation of continuously changing natural oral mouth conditions and (ii) the spatiotemporal biofilm profiling under dynamically controlled conditions without destructing the biofilm.

### Reproducible and stable biofilm generation with realistic abundances

In the drip flow reactor setup, the multispecies biofilm reproducibly develops over a time course of 3 days. The continuous supply of fresh medium causes the biofilm to be fully viable. Metabolic, structural, and compositional biofilm features remain stable from 3 to 6 days for the negative control experiment. The biofilm is dense—characterized by a high substratum coverage and biofilm volume, and a thickness of around 25–30 µm—and heterogeneous—characterized by the spread in measurements. However, over the time course of 3 days, averaged biofilm features (captured by 28 confocal images) are comparable, meaning that the *in vitro* biofilm model is reproducible and becomes globally more uniform.

Over the entire biofilm depth, the biofilm is dominated by commensal streptococcal species. These strains are initial colonizers with a fast growth (Supplementary file S3, Fig. A7), they consume primarily glucose (Supplementary file S3, Fig. A8), and can survive in both aerobic and anaerobic environments, resulting in its high numbers in the entire biofilm. The bridging organism *F. nucleatum* is also present in high numbers while the late colonizer and keystone pathogen, *P. gingivalis*-SNAP26 is present in low numbers within the biofilm, especially at the bottom. *F. nucleatum* is known to be able to co-aggregate with a wide variety of oral bacteria (43) and has a lower susceptibility to oxygen compared to *P. gingivalis* (5). As such, *F. nucleatum* might be more easily incorporated and can survive in less strict anaerobic environments in the biofilm, explaining its high numbers. The low numbers of *P. gingivalis*-SNAP26 probably result from competition with *F. nucleatum* for peptide consumption. In general, the prevalence and relative abundances of the involved species are similar to observed healthy conditions in the oral cavity as studied by Loozen et al. (6). The authors pictured the microbial prevalence and abundance of 20 oral bacteria in 6,308 patients. In periodontal health, streptococcal strains from the *gordonii* and *mitis* group occupy a large fraction of subgingival plaque. Although being associated with periodontal disease, *F. nucleatum* is also dominant while bacterial numbers of *P. gingivalis* are around four log values lower compared to the mentioned bacteria.

Metabolite profiles are consistent with the aerobic metabolism of the streptococcal strains (i.e., glucose and pyruvic acid consumption, and lactic acid and acetic acid production, Supplementary file S3, Fig. A8). Hydrogen peroxide is present in the biofilm but could not be measured quantitatively. Formic acid, propionic acid, and butyric acid, produced by *F. nucleatum* and *P. gingivalis*-SNAP26 in anaerobic conditions, hardly accumulate in the biofilm. The bacterial composition shows the presence of the anaerobic species; therefore, pointing at the occurrence of anaerobic micro-environments in the biofilm. Oxygen must be depleted either due to consumption by the streptococcal strains or diffusion limitations.

### L-arginine exerts multifaceted effects on periodontal biofilms

The application of L-arginine to the multispecies biofilm validates the usefulness of the model setup. In our study, a clinically relevant concentration (1.5% or 86 mM) of L-arginine is applied to a pre-formed healthy biofilm, and treatment responses are recorded over time. The biofilm mass and volume reduction within 24 hours of exposure are consistent with previous reports. L-arginine also lowered biofilm biomass and thickness of a single-species biofilm of *S. gordonii* when it was exposed to high arginine concentrations (50–500 mM) (35). This was accompanied with an altered biofilm architecture which was characterized by patchy biofilms with a higher roughness, also observed in our study. Contrarily, low arginine concentrations enhance biofilm development. The same concentration dependency was observed for a saliva-derived multispecies biofilm (37). While exposure to a low concentration of arginine (5 mM) increases the biofilm volume and thickness, exposure to high concentrations (*>*100 mM) significantly decreases the biofilm thickness and volume and increases the roughness. In our study, biofilm reductions are possibly caused by the decrease in total cell numbers in the biofilm. More specifically, cell numbers of *F. nucleatum* decrease with more than two log values, and biofilm disruptions could be addressed to reductions in green fluorescence. As *F. nucleatum* is known as a bridging organism, it largely shapes the biofilm community. Various studies also point at reduced amounts of extracellular polymeric substances (EPS), providing organization and cohesion within the biofilm matrix, due to arginine treatment as a possible underlying reason (37, 38, 44). L-arginine has the ability of breaking hydrogen bonds (45), which were proven to affect EPS interactions, resulting in a looser structure of the EPS layer (46). These disrupted interactions might also explain the greater penetration ability and the weakened *S. gordonii* biofilm observed by Kolderman et al. (37) and Gloag et al. (36), respectively. In our work, the EPS structure of the biofilm is not stained. Available EPS stainings are usually either toxic or destructive (not applicable for real-time studies) and target specific EPS structures, so ideally multiple dyes should be combined (47). Recently, optotracers are being used to monitor real-time EPS formation in a biofilm as these are non-toxic to cells (48, 49). The optotracer EbbaBiolight 680 (Ebba Biotech) was evaluated but is not suitable for staining the EPS structures produced by the selected oral strains in our study. Combining findings from our work and previous studies, we hypothesize that for lower arginine concentrations, the metabolic benefit of streptococcal strains enhances biofilm development, while for higher arginine concentrations, destabilizing effects on biofilm structure and co-aggregations become dominant.

Endpoint qPCR demonstrates the shift in bacterial biofilm composition after L-arginine treatment. The biofilm becomes less pathogenic, mainly caused by a significant reduction in *F. nucleatum* numbers. This, in turn, increases the relative abundances of *S. gordonii*-GFPmut3* and *S. sanguinis*-pVMCherry. The latter increase is confirmed by the structural biofilm analysis, showing an increased biofilm occupation by *S. sanguinis*-pVMCherry when exposed to L-arginine. We assume that reductions in pathogenic cell numbers are not addressed to increased hydrogen peroxide levels as L-arginine does not affect hydrogen peroxide production by streptococci (Supplementary file S3, Fig. A7). One potential reason for the observed compositional changes may be that *F. nucleatum* possesses the arginine-inhibitable adhesin, RadD, which is responsible for interacting with Gram-positive bacteria (50). It is thus likely that co-aggregation of *F. nucleatum* with the Gram-positive streptococcal strains is inhibited, hampering the retention/incorporation of *F. nucleatum* into the biofilm. We might even hypothesize that existing inter-species adherence is interrupted by the presence of L-arginine since *F. nucleatum* is initially present in high numbers in the biofilm. This shift is rather unexpected and not observed before. We speculate that the compositional changes contribute to a more heterogeneous spatial composition, which is represented in the increased variance in abundance of the green signal near the surface. Monoculture experiments with 1.5% L-arginine induce partial and complete growth inhibition for *F. nucleatum* and *P. gingivalis*-SNAP26, respectively (Supplementary file S3, Fig. A7). Only one other published work reports growth inhibition of *P. gingivalis*-SNAP26, which was in that case D-arginine (51). No indisputable conclusion on *P. gingivalis*-SNAP26 inhibition in the multispecies biofilm can be drawn from our data because the bacterial numbers of *P. gingivalis*-SNAP26 are close to the detection limit of qPCR, implying that this hypothesis needs further investigation. From a periodontal disease point of view, the decrease in *F. nucleatum* numbers is the most important. This effect is seen immediately. On a longer term, this can also positively impact *P. gingivalis*-SNAP26 numbers as these bacteria are closely (metabolically) interacting in a biofilm (52).

Compositional changes and the biofilm’s metabolism are interdependent. The presence of ornithine and ammonium points at the active metabolic conversion of L-arginine in *S. gordonii*-GFPmut3* and *S. sanguinis*-pVMCherry, increasing their relative abundance in the biofilm. Around 14% of the supplemented L-arginine is converted to ornithine, releasing ammonium. In the context of caries research, arginine metabolism neutralizes biofilm acidification, counteracting the deleterious effects of tooth demineralization (31, 32, 34). pH contributions are not considered in our study since pH fluctuations between healthy and periodontal diseased patients are less than one unit (53, 54). Besides ornithine and ammonium, other accumulated metabolite concentrations show small shifts during L-arginine treatment. Expected changes because of the lower total cell numbers are probably undetectable in the dense and viable biofilm.

All abovementioned effects of L-arginine on the multispecies biofilm are already effective after the first day of treatment, and structural shifts are most pronounced at the top where the biofilm is presumably more susceptible to incoming L-arginine. In turn, (i) the extracellular biofilm matrix is affected, reducing the overall incorporation capabilities and thus total cell numbers, (ii) co-aggregation between the streptococcal strains and *F. nucleatum* is disrupted and inhibited, reducing *F. nucleatum* numbers, and (iii) *S. gordonii*-GFPmut3* and *S. sanguinis*-pVMCherry can benefit from L-arginine as an additional resource, causing an increase in their relative percentages. Notwithstanding the instructiveness of the observed L-arginine effects, the *in vivo* persistent effectiveness might require significantly longer time periods. Multiple long-term caries trials only observed significant reductions in caries formation after 2 years of applying an arginine-containing toothpaste (55). Additionally, the use of an 8% arginine toothpaste for 8 weeks caused compositional changes in the saliva microbiome but not in the plaque microbiome of healthy individuals (56).

### Visualization of the multispecies community

Non-invasive, non-disruptive monitoring of the metabolically active biofilm community over a period of several days using confocal laser scanning microscopy requires fluorescent labeling of the involved micro-organisms. Selection of the fluorophores was done considering the technical constraints of the available lasers, the low oxygen conditions in the biofilm (e.g., required for maturation of the fluorescent protein), and the availability of genetic transformation tools. *F. nucleatum* could not be labeled because of the lack of genetic tools for this strain (57, 58), but leaving out *F. nucleatum* from the biofilm model was not an option because it is a well-known and abundant bridging micro-organism in the oral biofilm. Extensive analysis of *F. nucleatum*, however, showed a consistent autofluorescence, which was observed in more than hundred samples analyzed with flow cytometry (data not shown). Green fluorescence of *F. nucleatum* has been reported a few times under the excitation by visible blue light (59, 60), but the origin of fluorescence has never been addressed. Since all species in the drip flow biofilm reactor are viable (measured by vitality qPCR), autofluorescence of *F. nucleatum* will be captured on the green channel. We do not claim the exact quantification, but omitting this observation from the data interpretation would be mendacious.

To increase the number of differentially labeled species in the model, additional fluorochromes might be looked at. Very recently, Ponath et al. (61) developed several genetic tools for *F. nucleatum* including four distinct fluorescent proteins. Additionally, the oxygen-independent HaloTag (62) or CLIP-tag (63)—a SNAP-variant (64)—seems to be worth investigating. These tags are suitable with multiple synthetic dyes throughout the fluorescent spectrum.

The fact that *S. gordonii* and *S. oralis* are labeled identically was an intentional choice since only three colors/fluorescent labels could be distinguished on the available CLSM. Omitting one of the strains would not only simplify the biofilm model but also reduce the representativeness of the model.

### Conclusion

A setup to spatiotemporally monitor a periodontal biofilm was established. We showed that the drip flow reactor is a promising *in vitro* model system; flow is applied at the air-liquid interface, multiple bacterial species are used, stable, reproducible, and representative real-mouth conditions are guaranteed, and treatment approaches are applied on pre-formed biofilms. This study used the known prebiotic, L-arginine, as a proof-of-concept for a drip flow biofilm reactor that is the first one to spatiotemporally characterize a multispecies periodontal biofilm model. The multifaceted effects of L-arginine on the biofilm were validated and reproducible in the setup. The first effects are related to the amino acid itself. L-arginine has the ability of inhibiting growth and metabolism of the present pathogenic species. Moreover, the addition of L-arginine alters the mechanical and physical properties of the biofilm structure by affecting EPS and inhibiting *F. nucleatum* incorporation. Secondly, L-arginine serves as a substrate that is metabolized by *S. gordonii*-GFPmut3* and *S. sanguinis*-pVMCherry for energy and production of alkaline products, including ornithine and ammonium. Ornithine or ammonium itself does not seem to have a large impact on bacterial interactions in the biofilm.

## MATERIALS AND METHODS

### Bacterial strains and growth conditions

The bacterial strains used in this study are listed in Table 1. Strains were maintained on blood agar (Oxoid) plates supplemented with menadione (1 µg*/*mL), hemin (5 µg*/*mL), and sterile horse blood (5 vol/vol %, E&O Laboratories). Streptococcal strains were incubated in aerobic (5% CO_2_) conditions at 37°C. *F. nucleatum* and *P. gingivalis*-SNAP26 were grown anaerobically (5% CO_2_, 95% N_2_) at a temperature of 37°C. Precultures were incubated in brain heart infusion (BHI) broth (Becton Dickinson), supplemented with sodium pyruvate (2 g*/*L) for all strains, and supplemented with menadione (1 µg*/*mL) and hemin (5 µg*/*mL) for *F. nucleatum* and *P. gingivalis*-SNAP26. When a selection pressure was applied for selection or plasmid maintenance, erythromycin (5 µg*/*mL) was supplied to the cultures. *F. nucleatum* has natural resistance to erythromycin (39).

**TABLE 1:** Bacterial strains and plasmids used in this study

Strains (used in study)	Plasmid relevant protein/dye	Reference and kindly shared by the authors
*Streptococcus gordonii* DL1	pCM18 GFPmut3*	Hansen et al. (27)
*Streptococcus oralis* DSM 20627	pCM18 GFPmut3*	This study
*Streptococcus sanguinis* LMG14657	pVMCherry mCherry	This study
*Fusobacterium nucleatum* ATCC 10953	–^*a*^	–
*Porphyromonas gingivalis* ATCC 33277	SNAP26 SNAP-Cell TMR-Star	Nicolle et al. (22)

Strains (used for plasmid extraction)	Plasmid relevant protein/dye	Reference and kindly shared by the authors
*Streptococcus gordonii* DL1	pCM18 GFPmut3*	Hansen et al. (27)
*Streptococcus gordonii* CH1	pVMCherry mCherry	Vickerman et al. (19)

^*a*^ The hyphen '-' is used for non-fluorescently labeled strains.

### Plasmid DNA extraction and transformation

Plasmid DNA pCM18 and pVMCherry were extracted from *S. gordonii* DL1 and *S. gordonii* CH1, respectively, with the QIAprep Spin Miniprep Kit (QIAGEN) according to the manufacturer’s instructions. These plasmids, encoding the fluorescent proteins GFPmut3* and pVMCherry, are used to fluorescently label all the streptococcal strains via bacterial transformation. Modifications to facilitate cell lysis of Gram-positive strains were applied as described by Freitas et al. (65). *S. oralis* and *S. sanguinis*, which are naturally competent, were transformed using heat-inactivated horse serum (66, 67). In short, plasmid DNA was added to competent cells for 45 minutes after which DNAseI (10 µg*/*mL) and MgSO_4_ (10 mM) were supplied. After an additional 4 hours of incubation at 37°C, transformants were plated on tryptic soy agar (Sigma-Aldrich) supplemented with erythromycin (5 µg*/*mL), resulting in colonies of *S. oralis* pCM18 (GFPmut3*) and *S. sanguinis*-pVMCherry.

### Drip flow biofilm reactor setup

The drip flow biofilm reactor is used as a model setup because it allows biofilm growth at the air-liquid interface under a continuous laminar flow (16). Inspired by the widely applied flow cell (68), a glass slide is glued at the bottom of the adapted in-house drip flow reactor to allow real-time observation of the biofilm with the microscope. The drip flow biofilm reactor used in this study, shown in Fig. 1, was manufactured in-house from a polycarbonate rod (Vink). Dimensional drawings are presented in Supplementary file S5, Fig. A10. The substratum was a microscope borosilicate cover glass (24 mm × 50 mm), glued on the bottom of the reactor. To sterilize the reactor, 10 mL 0.5% sodium hypochlorite was added for 5 hours and completely washed away with sterile distilled water. Next, the setup was assembled with a syringe polytetrafluoroethylene (PTFE) filter (0.45 µm, Fischer Scientific), a mininert valve (VICI) for the beveled influent needle (22 gauge, 40 mM, BD Biosciences), and a sample port for the blunt effluent needle (17 gauge, 50.8 mm, Hamilton). Anaerobic conditions were established by purging with gas composed of 95% N_2_ and 5% CO_2_ and connecting a gas-filled balloon to the PTFE filter.

Before inoculation, precultures were mixed to a final OD_600nm_ of 0.1 for *S. gordonii*-GFPmut3*, *S. oralis*-GFPmut3*, *S. sanguinis*-pVMCherry, and *F. nucleatum* and an OD_600nm_ of 0.5 for *P. gingivalis*-SNAP26 [corresponding to cell numbers in the order of 5·10^8^ Geq/mL, 1·10^9^ Geq/mL, 3.5·10^7^ Geq/mL, 1·10^9^ Geq/mL, and 1.5·10^9^ Geq/mL, respectively (determined with qPCR afterwards)], in supplemented modified Brain Hearth Infusion (smBHI). This medium contains modified BHI (69) supplemented with 2 g*/*L sodium pyruvate, 3.4 g*/*L potassium dihydrogen phosphate, 4.4 g*/*L dipotassium hydrogen phosphate, and 2.5 µg*/*mL erythromycin. The five-species mixture was connected to the drip flow reactor and circulated for 8 hours at 37°C with a flow rate of 0.11 mL*/*minute under anaerobic conditions. This recirculation phase allowed initial bacterial growth and biofilm attachment to the glass slide. Afterward, 1:2 diluted supplemented mBHI (1:2 smBHI) was continuously fed to the biofilm in aerobic conditions (5% CO_2_) at the same flow rate. Daily metabolic analysis and confocal imaging combined with end-point qPCR measurements return metabolite consumption and production profiles (the difference in metabolite concentrations at the in- and outlet represents the metabolism of the whole biofilm), and structural and compositional biofilm features, respectively.

**Fig 10:**
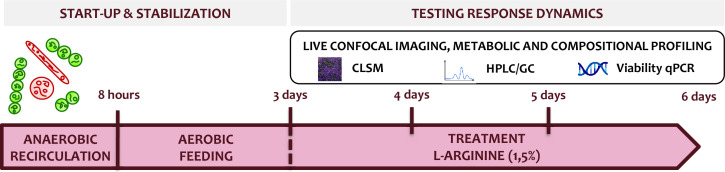
Timeline for testing L-arginine response dynamics. Starting from a mature, 3-day developed biofilm, L-arginine is continuously supplied. Response effects are measured every day for three consequent days.

Treatment effects of L-arginine are studied according to the experimental timeline in Fig. 10. Starting from a stable and reproducible biofilm of 3 days, L-arginine (1.5%) is added to the medium (1:2 smBHI, pH of 6.7) for another 3 days. The analyses performed right before L-arginine addition (day 3) serve as a reference time point to compare all measurements with. Three biological replicates are performed. The negative control is fed with half strength smBHI without the addition of L-arginine.

### Metabolite concentrations

Extracellular metabolite concentrations were determined in the effluent (collected over a period of 20 minutes) of the drip flow reactor. Sugars and organic acids in the samples were measured on an Agilent 1200 HPLC with an injection volume of 20 µL. An Aminex HPX-87 column was kept at 40°C and eluted with H_2_SO_4_ (5 mM) at a flow rate of 0.6 mL/minute. Components were detected with an ultraviolet or refractive index detector. Amino acids (free and protein-bound) in the samples were measured with GC-FID. First, effluent samples were hydrolyzed with HCl (6 M) supplemented with thioglycolic acid (4%) in a nitrogen environment for 22 hours at 105°C. Next, samples were treated with the EZ:faast Kit (Phenomenex) for protein hydrolysates according to the instruction manual. Amino acid derivatives were determined on a GC-FID (Perkin Elmer) equipped with a Zebron ZB-AAA column. Ammonium was measured on a 930 Compact Ion Chromatograph Flex (Metrohm) equipped with a Metrosep C6-150/4.0 column and a Metrosep C6 Guard/4.0 column. The eluent was composed of HNO_3_ (1.7 mM) and dipicolinic acid (1.7 mM), and components were measured with a conductivity detector.

Hydrogen peroxide was measured with the Amplex Red Hydrogen Peroxide/Peroxidase Assay Kit (Invitrogen). Briefly, hydrogen peroxide reacts stoichiometrically in a 1:1 relation with Amplex Red (N-acetyl-3,7-dihydroxyphenoxazine), catalyzed by horseradish peroxidase (HRP), to the red-fluorescent oxidation product, resorufin. For a qualitative verification of H_2_O_2_ production by the biofilm, the Amplex Red solution (containing 173 µM Amplex Red, 0.2 U/mL HRP, and 0.5 mM glucose diluted in PBS) was added to the drip flow reactor after removal of spent medium. After 10 minutes of incubation, the fluorescence was measured with CLSM with an excitation at 552 nm and emission detection at 570–610 nm. For this experiment, all streptococcal strains were labeled with GFPmut3* to avoid interference between resorufin and mCherry.

### Bacterial DNA extraction and quantitative polymerase chain reaction

Viability qPCR with the membrane-impermeable DNA dye propidium monoazide (PMA) was used to determine live and total bacterial numbers in the biofilm (70, 71). Biofilms were disrupted and detached from the glass substrate by trypsinization with 0.05% Trypsin-EDTA (Gibco) while being shaken at 150 rpm for 45 minutes at 37°C. The remaining biofilm pellet was resuspended in PBS from which 95 µL was treated with propidium monoazide (PMAxx Biotium) in a final concentration of 100 µM. Samples were gently shaken on the rock shaker for 5 minutes in the dark, after which the dye was activated by exposing the samples to the Glo-Plate Blue Led Illuminator (Biotium) for 15 minutes. Untreated and PMAxx-treated samples were centrifuged for 10 minutes at 5,000 × *g*. Bacterial DNA from the pelleted biofilm was extracted using the QIAamp Mini Kit (Qiagen) in accordance with the manufacturer’s instructions.

Bacterial numbers were quantified by probe-based TaqMan qPCR assays according to Van Holm et al. (72) using a CFX96 real-time system (Bio-Rad) with the associated software CFX Manager (version 3.1). TaqMan reactions contained 5 µL template DNA, 1 µL primers and probes, 12.5 µL Takyon No Rox probe master mix dTTP (Eurogentec), and 4.5 µL distilled water. Final bacterial cell numbers were expressed as genome equivalents per milliliter. Sequences and concentrations of the bacterium-specific primers and probes were given in Supplementary file S5, Table A2.

### CLSM and image analysis

Confocal laser scanning microscopy was used to analyze the three-dimensional structure of the biofilm. Prior to microscopy analysis, 1 mL medium supplemented with SNAP-Cell TMR Star (0.6 µM, New England Biolabs) was supplied to the drip flow reactor for 30 minutes at 37°C to stain *P. gingivalis*-SNAP26. Afterward, the medium was pumped out, and the biofilm was analyzed with a Leica TCS SP8 inverted confocal laser scanning microscope equipped with a 63× glycerol immersion objective, and a 488-nm and 552-nm solid-state laser. Hybrid detectors were set to 495–545 nm (green), 620–660 nm (pink), and 560–600 nm (red) for detecting GFPmut3*, mCherry, and TMR-Star, respectively. Confocal images were obtained in sequential scanning mode with excitation of each laser separately to avoid spectral overlap between mCherry and TMR-Star. At each time point, 28 images (184.52 µm × 184.52 µm) were taken at specific locations excluding the left and right 15 mm outer regions as shown in Supplementary file S5, Fig. A11. In each of the seven white boxes, four Z-stacks were generated with a distance of 1 µM. The 28 z-stacks have the same location for each time point.

Images were analyzed with the software BiofilmQ (73) to determine biofilm parameters including roughness coefficient, mean thickness, biofilm volume, and substratum coverage. Images were first thresholded into fore- and background with the Otsu method (74) separating pixels in three classes for GFPmut3* and mCherry, and in two classes for SNAP Cell-TMR Star. To determine global biofilm parameters, images were filtered with a Kernel, convolution matrix of 3 by 3 before calculations were performed. Individual color percentages at each layer of the biofilm were calculated on thresholded, unfiltered images with a self-written Matlab script derived from the source code.

### Models and statistical analysis

MATLAB R2021b (MathWorks) was used for creating images and fitting mathematical models. Box plots were created with the function *daboxplot* (https://github.com/frank-pk/DataViz).

Comparisons between the negative control and treatment experiment were performed using a linear mixed model in R [version 4.2.2 (https://www.R-project.org/)]. The assumptions of the model were checked by a normal quantile plot of the residual values and a residual dot plot. For time-dependent data (i.e., accumulated metabolite profiles and biofilm features derived from image analysis), differences between the negative control and treatment at each time point are compared to differences at time point 72 (i.e., reference point). For metabolite profiles and qPCR data, *P*-values were corrected for simultaneous hypotheses according to Sidak (groups of one factor are compared with each group of the other factor). *P*-values below 0.05 were considered to be statistically significant.

## Data Availability

The data that support the findings of this study are included in the manuscript, the supplementary information, or available from the corresponding author upon reasonable request.

## SUPPLEMENTAL MATERIAL

Supplementary files S1 to S5 aem.01081-23-s0001.pdf Supplementary files S1 to S5 containing all additional text, tables, and figures.

ASM does not own the copyrights to Supplemental Material that may be linked to, or accessed through, an article. The authors have granted ASM a non-exclusive, world-wide license to publish the Supplemental Material files. Please contact the corresponding author directly for reuse.
