# Supplementary material for: Spatiotemporal monitoring of a periodontal multispecies biofilm model: demonstration of prebiotic treatment responses
Source: Appl Environ Microbiol. 2023 Sep 28;89(10):e01081-23. doi: 10.1128/aem.01081-23 (PMC10617495; doi:10.1128/aem.01081-23)
Supplement: Supplementary files S1 to S5 — containing all additional text, tables, and figures. [file aem.01081-23-s0001.pdf]

Supplementary file S1

Fluorescent labels and fluorescence compatibility

The fluorescently labelled *S. gordonii*-GFPmut3*, *S. oralis*-GFPmut3*, *S. sanguinis*-pVMCherry, and *P. gingivalis*-SNAP26 are used in this study (Table 1). On the one hand, the available lasers and detectors determined the number of detectable colors and selectivity in detection (see Materials and Methods). On the other hand, we considered available fluorescent labels for the oral bacteria and suitable for oxygen-restricted conditions. For oral streptococcal strains, most studies use fluorescent expression in the green and red regions (1, 2, 3,4), limiting the specific visualization of more than two oral streptococcal strains. Given the fluorescence compatibility and thus specific visualization of *S. gordonii*-GFPmut3* and *P. gingivalis*-SNAP26 in a bispecies biofilm demonstrated by Nicolle et al. (5), these strains were selected for the 5-species model. Both labels return satisfactory fluorescence under anaerobic conditions (3, 5). In addition, the plasmid pCM18 (GFPmut3*) (3) was extracted and introduced in *S. oralis* yielding bright and stable green fluorescence. Finally, the plasmid pVMCherry with streptococcal codon-optimized mCherry from Vickerman et al. (1) was transformed in *S. sanguinis* (this study). The use of a fluorescent protein in *F. nucleatum* was completely hampered by the lack of universal genetic tools and optimized plasmids (6, 7) at the time of the study. The recently published work of Ponath et al. (8) was not yet available.

Laser and detector settings of the confocal microscope were optimized to discriminate between the different fluorescent signals expressed by the bacteria. Figure A1 shows the fluorescence compatibility of the fluorochromes in a liquid culture containing the five oral bacteria. Overlapping the three colors with a bright field perspective in Figure A1a gives a good discrimination between *S. gordonii*-GFPmut3* and *S. oralis*-GFPmut3* in green, *S. sanguinis*-pVMCherry in magenta, and *P. gingivalis*-SNAP26 in red. These bacteria do not emit autofluorescence. Being relatively small compared to the streptococcal strains, *P. gingivalis* is harder to visualize by eye, but easily detectable by image analysis software. Some bright field elements are not colored because not all cells are well-aligned into the specific z-stack, weakening the fluorescent signal. For the elongated, rod-shaped *F. nucleatum*, autofluorescence could be observed in the green detector channel, illustrated by the black circle in Figure A1a. This autofluorescence was confirmed by

flow cytometry (Figure A2). The signal was hardly captured in the green detector channel for an early exponential culture of *F. nucleatum*, but when reaching the stationary phase (more related to biofilm maturation), microscopy and flow cytometry data show increased fluorescent intensities (Figures A3 and A2). After performing the image thresholding and filtering steps on the separate channels in BiofilmQ (9), the quantified fluorescence intensities in the GFPmut3*, mCherry, and TMR-Star channels are presented in Figures A1b, A1c, and A1d, respectively. They align very well with the bacterial cells observed in bright field view and show hardly overlap between the channels.

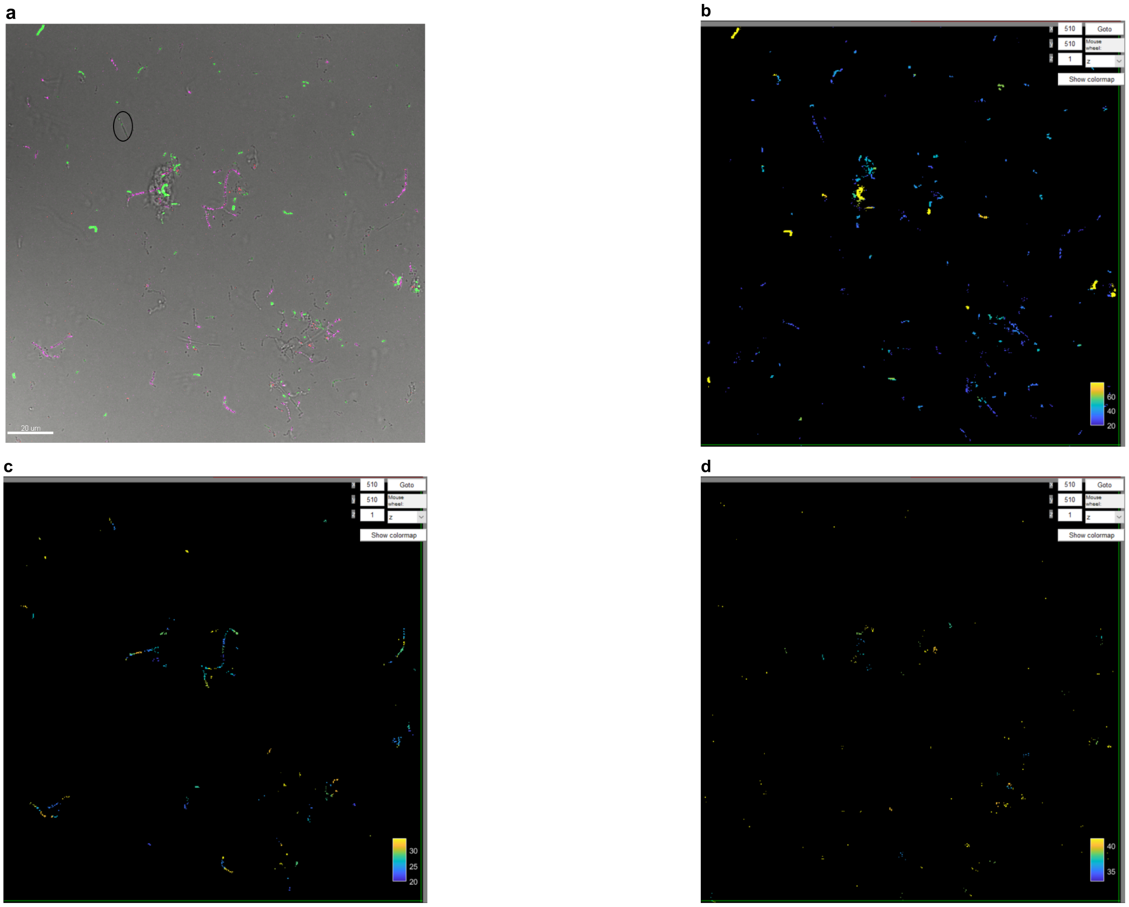

Figure A1: Fluorescence specificity and compatibility of the fluorochromes GFPmut3* (green), mCherry (magenta), and SNAP-Cell TMR Star (red) in a liquid culture containing *S. gordonii*-GFPmut3*, *S. oralis*-GFPmut3*, *S. sanguinis*-pVMCherry, *F. nucleatum*, and *P. gingivalis*-SNAP26. Fluorescence signals for all laser-detector settings are shown. Images are thresholded with the Otsu method. **a.** Overlap of all fluorescent channels and the bright field channel. **b, c, d.** Fluorescent intensities in the GFPmut3*, mCherry, and SNAP-Cell TMR Star detector channels, respectively, after image analysis with the BiofilmQ software.

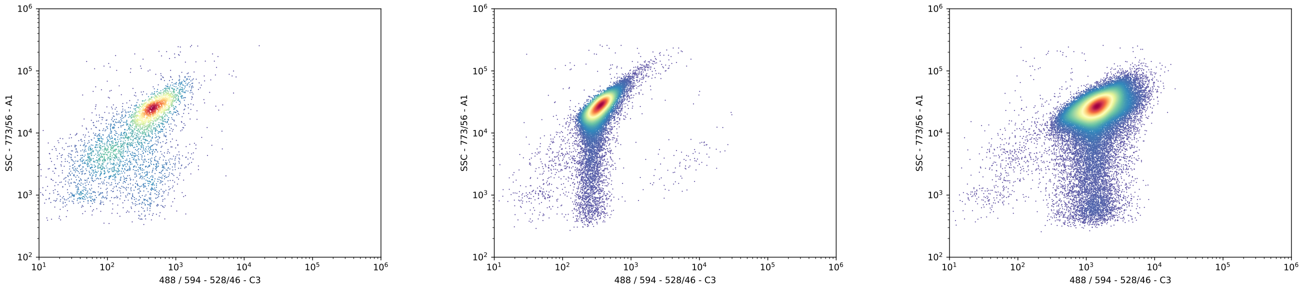

Figure A2: Flow cytometry graphs of a liquid culture of *F. nucleatum* over time. Density plots of side scatter versus the green channel (528 nm, 46 nm bandwidth) are shown. Graphs represent 2 (**a**), 8 (**b**), and 24 hours (**c**) of growth, respectively.

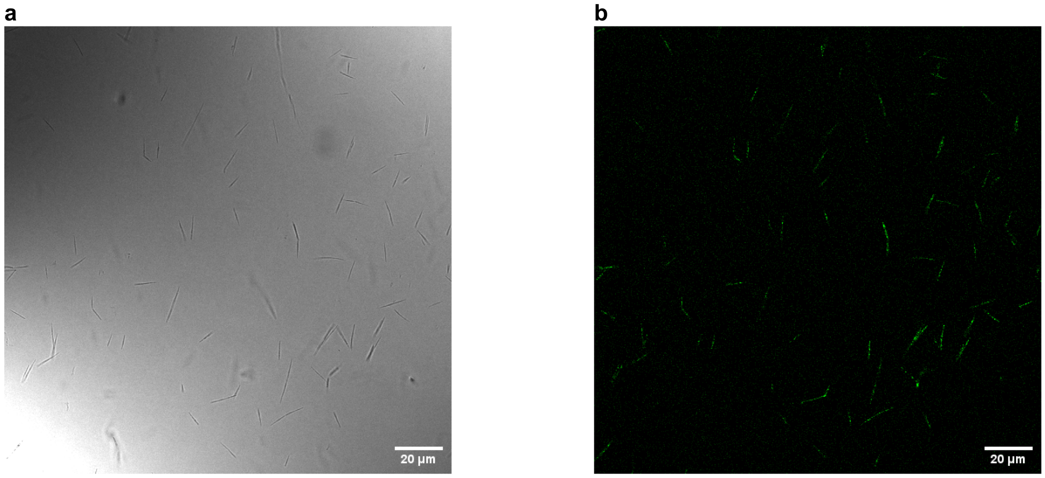

Figure A3: Autofluorescence signal of a liquid, mature grown culture of *F. nucleatum*. **a.** Bright field channel. **b.** Green fluorescent channel.

Flow cytometry Flow cytometry was used to investigate the autofluorescence of *F. nucleatum* during its growth. 1 mL of a liquid culture was centrifuged at 5000 $\times$g for 10 minutes and the pellet was resuspended in 0.22 μm-filtered PBS to obtain a concentration in the range between $10^7 - 10^8$ cells/mL. Samples were analyzed with the Amnis® CellStream® (Luminex) at a flow rate of 3.66 μL/min. Side scatter is measured with a 785 nm laser (40 %). *F. nucleatum* was excited by the 488 nm laser (100 %) and autofluorescence was detected with a charge-coupled device (CCD) camera using the filter stack 528 nm with a bandwidth of 46 nm, referred to as the green channel. Density plots of side scatter versus the green channel are presented. Data is gated on the aspect ratio, calculated as the ratio of the length of the minor cell axis over the length of the major cell axis.

Supplementary file S2

Additional information to Results

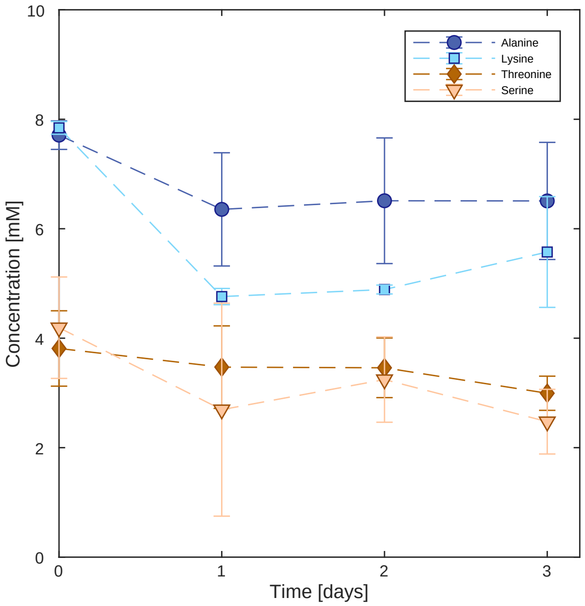

Figure A4: Amino acid (free and protein-bound) concentrations in the effluent of the drip flow reactor reflecting metabolic activity of the biofilm. Averages and standard deviations of two biological replicates are shown. Relevant amino acids are selected to be shown.

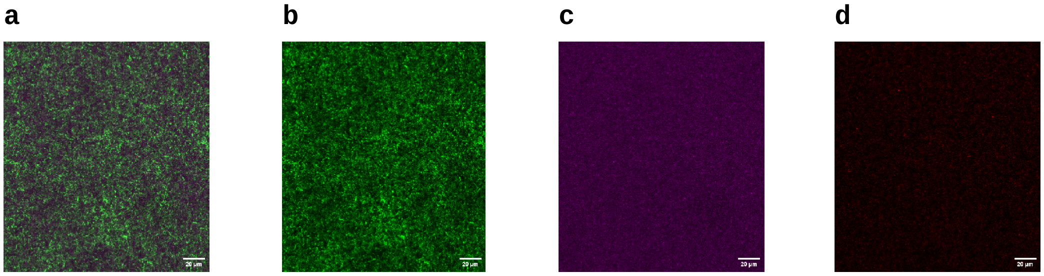

Figure A5: **a.** Fluorescence signals for a qualitative measurements of hydrogen peroxide in the drip flow biofilm. **b.** *S. gordonii*-GFPmut3*, *S. oralis*-GFPmut3*, *S. sanguinis*-GFPmut3*, and *F. nucleatum* are detected in the green channel. **c.** In the presence of hydrogen peroxide and HRP, the Amplex Red reagent reacts into resorufin, a red-fluorescent oxidation product, seen in the magenta channel. **d.** *P. gingivalis*-SNAP26 is detected in the red channel. Images are thresholded with the Otsu method. Maximum intensity projection in the software Imaris (bitplane) renders a 2D representation of the stack.

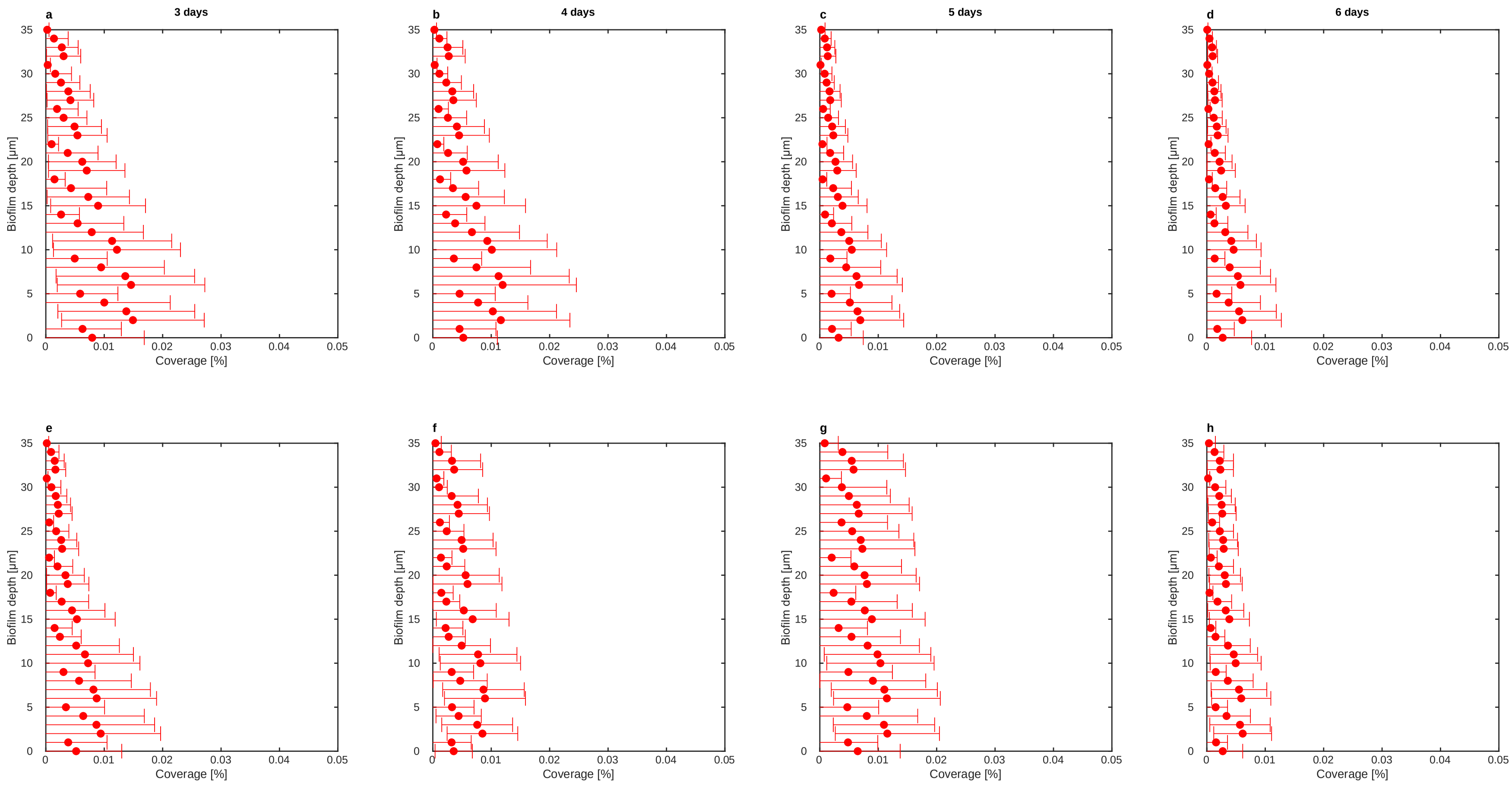

Figure A6: Depth profiles illustrating the color coverages at each height of the biofilm (and thus the spatial composition) over time for the negative control (top row) and L-arginine treatment (bottom row). The x-axes are zoomed in to see the abundance as a function of the biofilm depth for *P. gingivalis*-SNAP26. Time point measurements are performed on 28 confocal images per three biological replicates. Statistics are summarized in Supplementary table A1.

Supplementary file S3

Growth and metabolic characterization

Interpretation of biofilm metabolite data requires knowledge of individual species growth, metabolic activity, and the effect of L-arginine on growth and metabolic products. Although planktonic growth conditions result in unlimited substrate availability, typical patterns in conversion of substrates into metabolic products are expected to align with diffusion-limited growth conditions in a biofilm. Given the possible interactions between different strains in close proximity in the biofilm, an interaction model on the basis of competition of substrates and metabolic cross-feeding is drawn.

Materials & Methods Batch experiments were performed to characterize the growth and metabolite profiles of the individual bacterial species in a liquid medium. A parallel bioreactor system (DASGIP Eppendorf) was used. Experiments are performed in 750 mL BHI, supplemented with sodium pyruvate (2 g/L, to be consistent between suppliers of BHI) and 150 μL antifoam. When growing *F. nucleatum* and *P. gingivalis*-SNAP26, hemin (5 μg/mL) and menadione (1 μg/mL) were supplemented to the medium. The temperature, agitation speed, and gas flow rate were set at 37°C, 300 rpm, and 1 vvm (volume of gas per volume of medium per minute), respectively. The pH was kept at 6.7 $\pm$ 0.1 by automatically dosing H_2SO_4 (2M) and NaOH (3M). The bioreactor was inoculated with a bacterial preculture, diluted in fresh BHI medium. The streptococcal strains were grown in aerobic (5 % CO_2) conditions while *F. nucleatum* and *P. gingivalis*-SNAP26 were grown in anaerobic (5 % CO_2, 95 % N_2) conditions. Bacterial growth was determined by off-line spectrophotometric measurements at 600 nm (GENESYS™ 10S UV-Vis Spectrophotometer). Extracellular metabolite concentrations were determined in the supernatant (after centrifugation at 5000 $\times$g for 10 min) by off-line chromatography as described in Materials and Methods.

Hydrogen peroxide was measured in phosphate buffered saline (PBS, at pH 7.4) according to Verspecht et al. (10). The use of PBS avoids the Fenton reaction with medium components (11). In short, 5 mL samples were taken, centrifuged at 4°C and 5000 $\times$g for 10 min, and the supernatant was discarded. The cell pellet was washed twice with ice-cold PBS, resuspended in 10 mL PBS supplemented with 0.5 mM glucose, and aerobically incubated at 37°C and 200 rpm. After 2 hours,

5 mL of the culture was sampled and centrifuged (4°C, 5000 ×g, 10 min). The supernatant was filter sterilized, snap frozen in liquid nitrogen, and stored at -20°C for further analysis. Hydrogen peroxide concentrations were finally determined with the Amplex™ Red Hydrogen Peroxide/Peroxidase Assay Kit (Invitrogen) according to the manuals instructions. The fluorescence signal was measured in a Tecan Infinite 200 PRO, with 530 nm excitation and 590 nm emission detection. Using this complex protocol, the intrinsic capacity of the streptococcal strains to produce hydrogen peroxide could be quantified.

Results Monoculture experiments are performed with and without the addition of 1.5 % L-arginine. Growth curves are shown in Figure A7a and A7b. Figure A7d depicts the metabolic interaction network, derived from metabolite concentration profiles (Figures A8 and A9) and co-culture experiments (data not shown). Amino acids and peptides are present in the medium and contribute to growth. Total amino acid (i.e., free and protein-bound) concentrations are measured because the anaerobic strains prefer the usage of peptides to amino acids (12).

The streptococcal strains grow relatively fast (maximum OD in less than a day) while *F. nucleatum* and *P. gingivalis*-SNAP26 are slow growers. The streptococcal strains consume glucose and pyruvic acid as substrates for growth while producing organic acids, i.e., acetic acid, lactic acid and formic acid. *F. nucleatum* first converts pyruvic acid into biomass, acetic acid, lactic acid and formic acid. Upon depletion of pyruvic acid, glucose, peptides, and formic acid are converted into biomass, lactic acid and butyric acid. *P. gingivalis*-SNAP26 primarily uses peptides for growth and subsequently, pyruvic acid is consumed to produce organic acids including propionic acid and butyric acid.

Hydrogen peroxide acts as an antimicrobial, limiting the growth of pathobionts (13), and was therefore measured (10). Quantitative concentration profiles of the hydrogen peroxide production potential during growth of the streptococcal strains are determined (Figure A7c). These have not been reported in any study before. All streptococcal strains start producing hydrogen peroxide in the exponential growth phase and continue production in the stationary phase with concentrations up to 0.28 mM, 1.28 mM, and 0.32 mM for *S. gordonii*-GFPmut3*, *S. oralis*-GFPmut3*, and *S. sanguinis*-pVMCherry, respectively, at a similar average final OD of 1.25. Under aerobic conditions, pyruvate oxidase (SpxB) catalyzes the generation of hydrogen peroxide, carbon dioxide, and acetyl phosphate.

The latter is further metabolized into acetate (12, 14, 15). With this in mind, the very high hydrogen peroxide production observed by *S. oralis*-GFPmut3* might be directly related to the observed higher pyruvate uptake rate and acetate production rate compared to *S. gordonii*-GFPmut3* and *S. sanguinis*-pVMCherry.

Growth and metabolism of the community members are impacted by the addition of L-arginine, as shown in Figure A7a and A7b. *S. gordonii* GFPmut3* and *S. sanguinis*-pVMCherry reaches higher optical density values (A7a). L-arginine is metabolized into biomass and converted into alkaline products, including ornithine (Figure A9). Ornithine results from the degradation of citrulline in the arginine deiminase system (12). *S. oralis*-GFPmut3* does not metabolize L-arginine and consequently has no growth benefit or ornithine production. Hydrogen peroxide production is not altered as a result of the addition of L-arginine. Contrarily to the streptococcal strains, growth and metabolic activity are partly impaired for *F. nucleatum*, and *P. gingivalis*-SNAP26 is even completely inhibited by the presence of L-arginine.

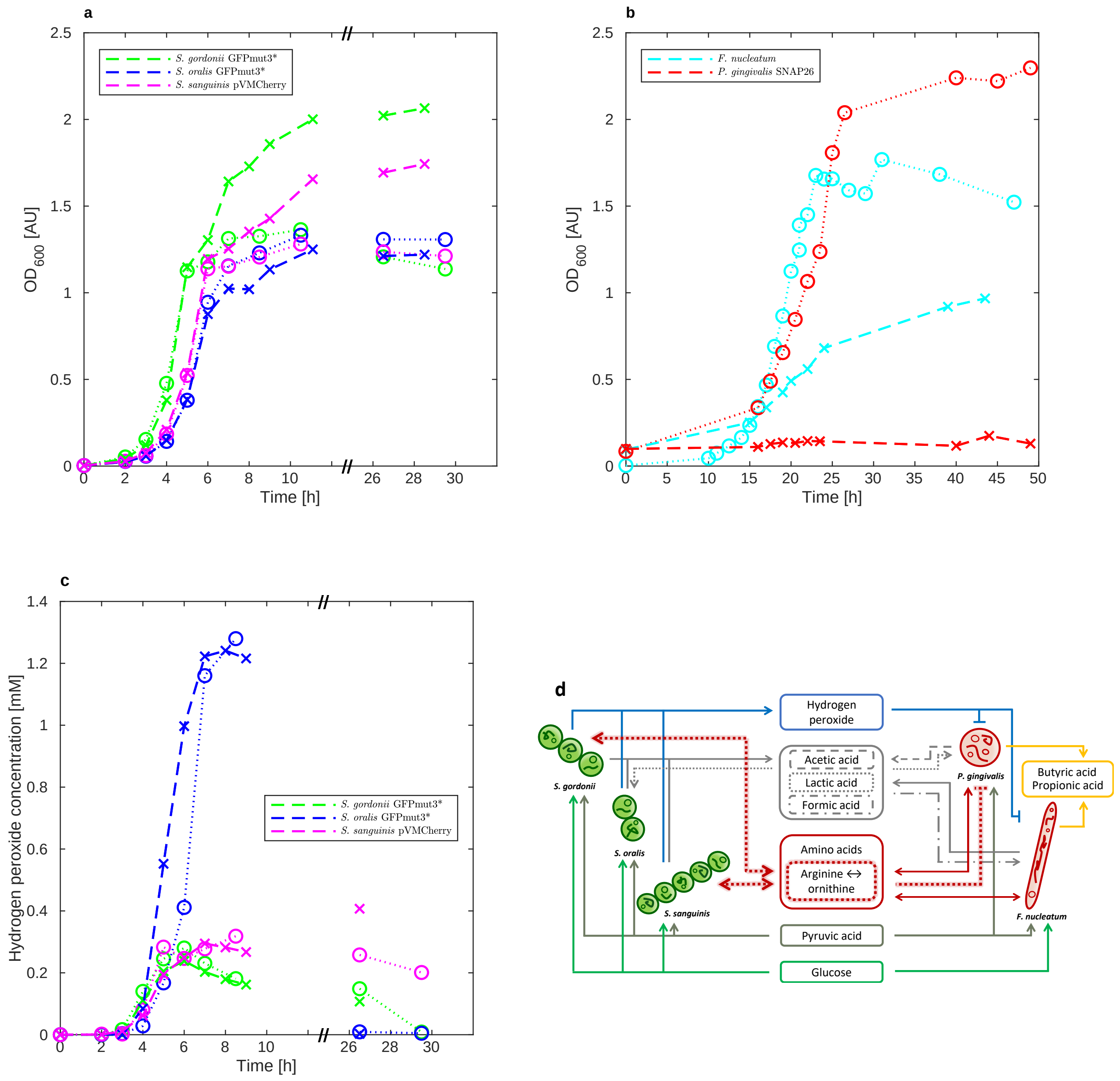

Figure A7: Growth and metabolic characterization of the community members. **a, b.** Optical density over time of monoculture experiments. **c.** Hydrogen peroxide production potential over time for the streptococcal strains. Dots '○' represent experiments in BHI medium supplemented with sodium pyruvate. Crosses '×' represent experiments where L-arginine is supplemented. **d.** Metabolic interaction network of the community. Arrows pointing at metabolites represent production while arrows pointing at the bacteria represent consumption. Blocked lines represent inhibition of the bacteria. Red dashed lines show L-arginine metabolization by *S. gordonii*-GFPmut3* and *S. oralis*-GFPmut3* resulting in higher cell numbers and the inhibition of *P. gingivalis*-SNAP26 in the presence of 1.5% L-arginine.

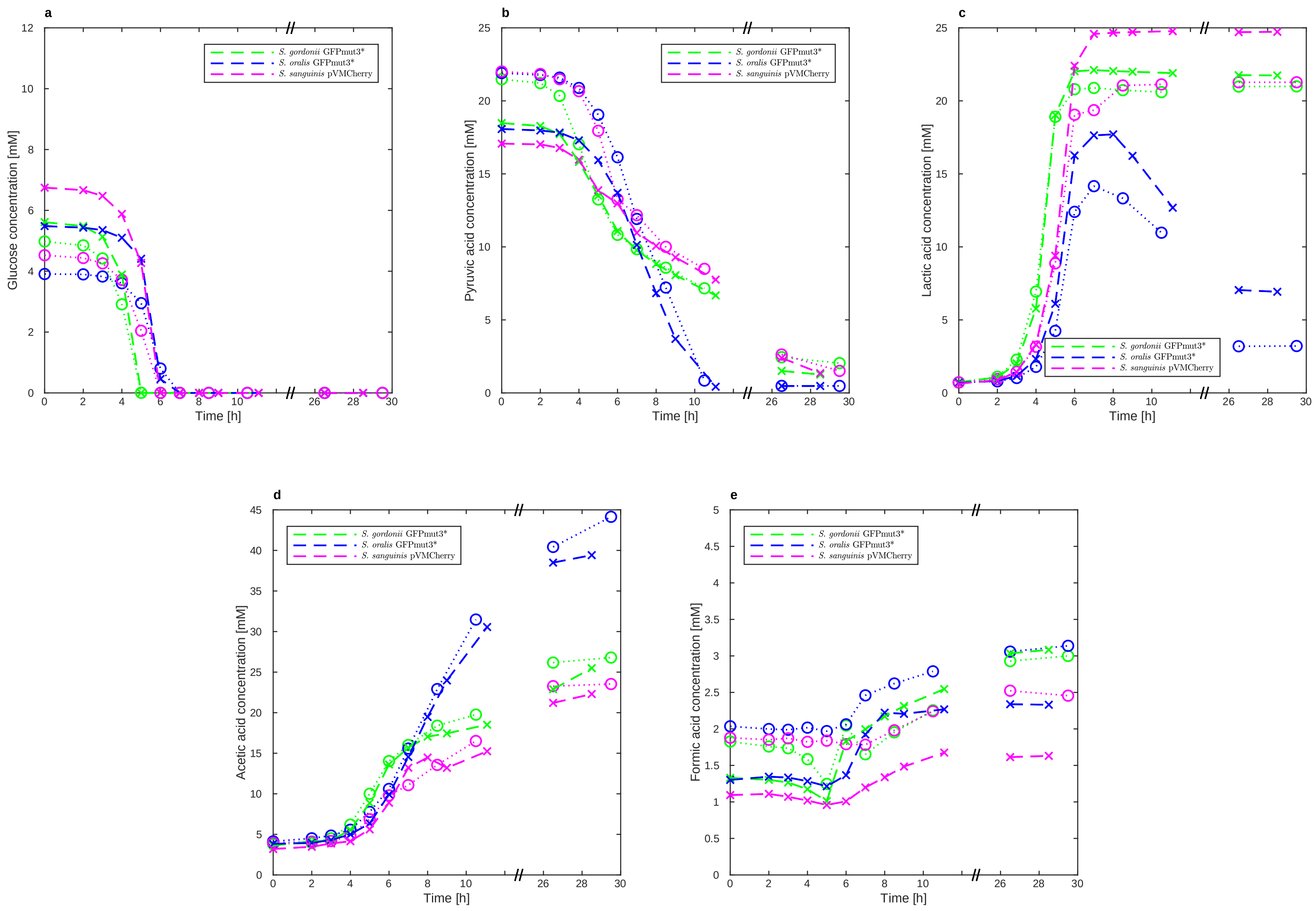

Figure A8: Glucose and organic acid concentration profiles of the community members during growth. Dots '○' represent experiments in BHI medium supplemented with sodium pyruvate. Crosses '×' represent experiments where L-arginine is supplemented.

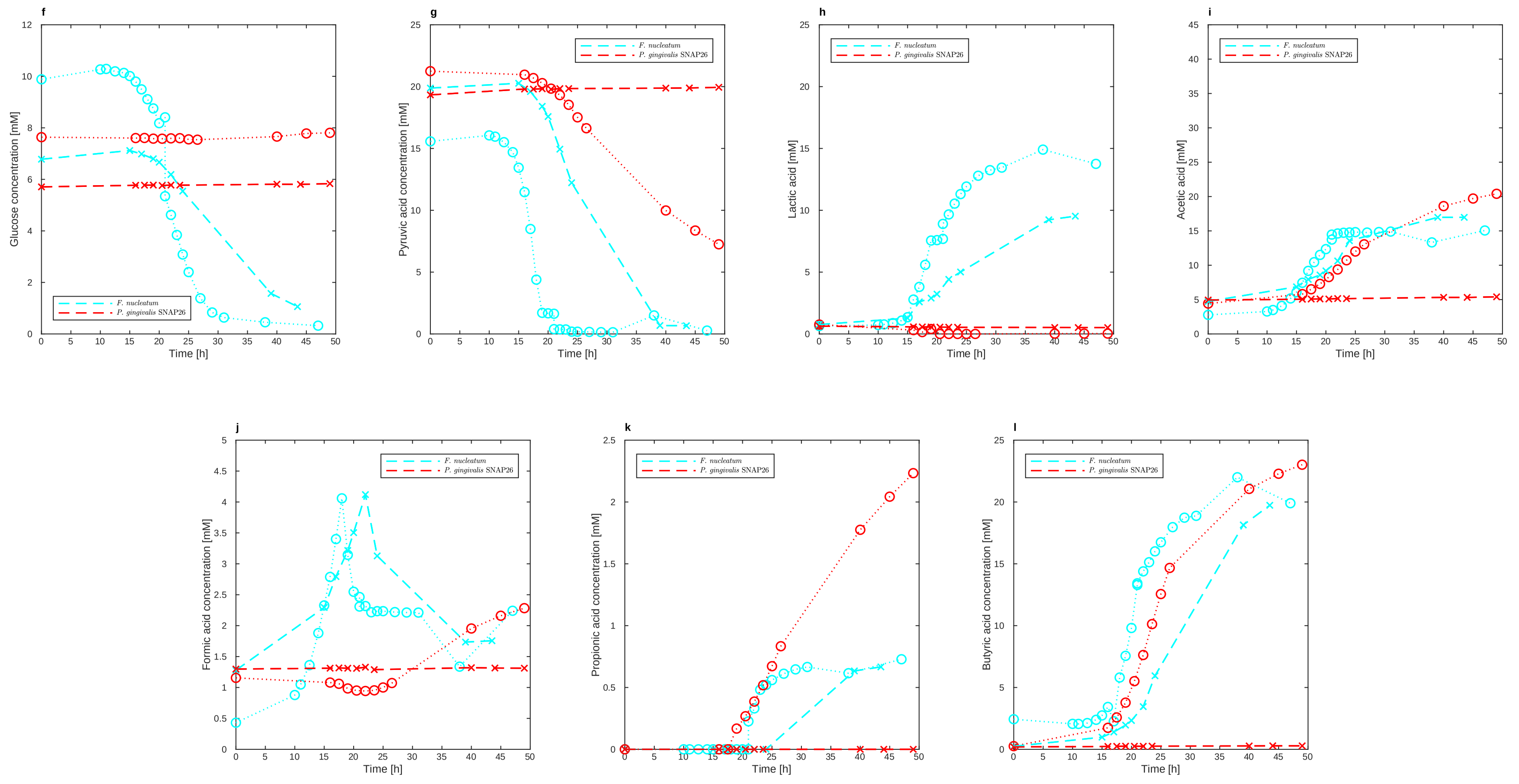

Figure A8 (cont.)**:** Glucose and organic acid concentration profiles of the community members during growth (continued). Dots ’○’ represent experiments in BHI medium supplemented with sodium pyruvate. Crosses ’×’ represent experiments where L-arginine is supplemented.

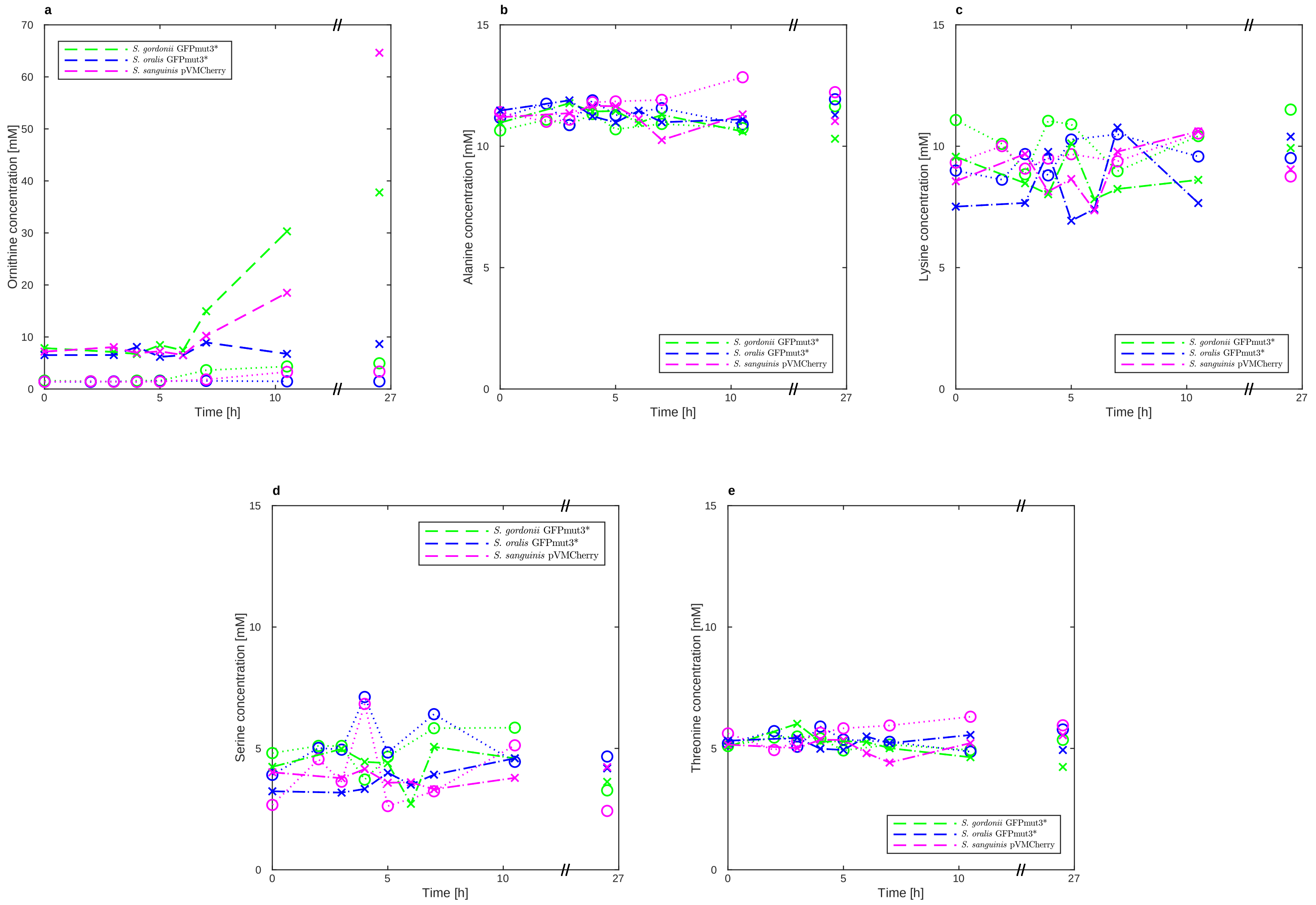

Figure A9: Amino acid (free and protein-bound) profiles of the community members during growth. Dots '∘' represent experiments in BHI medium supplemented with sodium pyruvate. Crosses '×' represent experiments where L-arginine is supplemented. Relevant amino acids are selected to be shown.

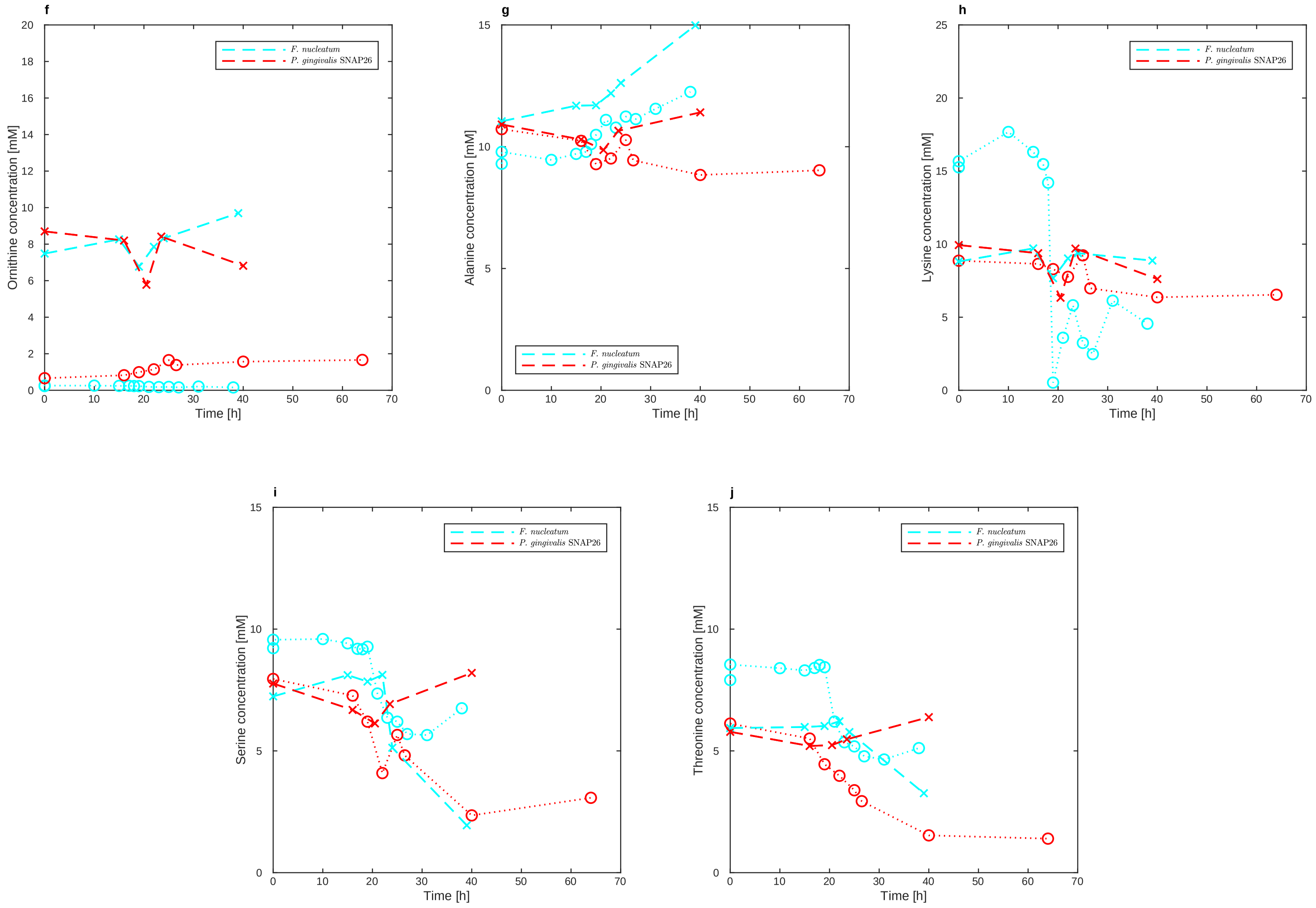

Figure A9: Amino acid (free and protein-bound) profiles of the community members during growth (continued). Dots '○' represent experiments in BHI medium supplemented with sodium pyruvate. Crosses '×' represent experiments where L-arginine is supplemented. Relevant amino acids are selected to be shown.

Supplementary file S4

Table A1: Statistics of the depth profiles which are shown in Figure 8. Differences between the negative control and treatment at each time point are compared to differences at time point 72 (i.e., reference point).

No significant interaction between the biofilm depth and treatment or time

**Parameter**	**Fluorescent color**	**Timepoint [hours]**	**p-value**
Average	mCherry	96	< 0.001 ***
Average	mCherry	120	0.00533 **
Average	mCherry	144	0.99992
Average	SNAP26	96	0.723
Average	SNAP26	120	$< 1 \cdot 10^{-5}$ ***
Average	SNAP26	144	$< 1 \cdot 10^{-5}$ ***
Standard deviation	mCherry	96	$< 1 \cdot 10^{-4}$ ***
Standard deviation	mCherry	120	$< 1 \cdot 10^{-4}$ ***
Standard deviation	mCherry	144	0.00104 **
Standard deviation	SNAP26	96	0.066
Standard deviation	SNAP26	120	< 0.001 ***
Standard deviation	SNAP26	144	< 0.001 ***

Significant interaction between the biofilm depth and treatment or time

**Parameter**	**Fluorescent color**	**Timepoint [hours]**	**Biofilm depth [μm]**	**p-value**
Average	GFPmut3*	96	0	0.0435 *
Average	GFPmut3*	120	0	0.0755
Average	GFPmut3*	144	0	0.9426
Standard deviation	GFPmut3*	96	35	0.0576
Standard deviation	GFPmut3*	120	35	0.0464 *
Standard deviation	GFPmut3*	144	35	0.0363 *

No significant interactions implies analysis over all depth data.
Data for all depth layers is available upon request.

Supplementary file S5

Additional information to Materials and Methods

Table A2: Primer and probe sequences and concentrations for quantification of bacterial numbers by (vitality) qPCR

Bacterium Target gene	Primers and probes 5'-3' sequence	Final conc.	Amplicon length
S. gordonii *gtfG*	**Forward primer:** GAAGAACTGGGTAGCGATTGCT	400 nM	177 bp
	**Reverse primer:** GTTAGCTGTTGGATTGGTTGCC	400 nM	
	**Probe:** FAM-AGAACAGTCCGCTGTTCAGAGCAA-BHQ-1	100 nM	
S. oralis *gtfR*	**Forward primer:** ACCAGCAGATACGAAAGAAGCAT	400 nM	229 bp
	**Reverse primer:** AGGTTCGGGCAAGCGATCTTTCT	400 nM	
	**Probe:** FAM-AAGGCTGCTGTTGCTGAAGAAGT-BHQ-1	100 nM	
S. sanguinis *gtfP*	**Forward primer:** CAAAATTGTTGCAAATCCAAAGG	600 nM	75 bp
	**Reverse primer:** GCTATCGCTCCCTGTCTTTGA	600 nM	
	**Probe:** FAM-AAAGAAAGATCGCTTGCCAGAACCGG-BHQ-1	100 nM	
F. nucleatum 16S rRNA	**Forward primer:** GGATTTATTGGGCGTAAAGC	300 nM	191 bp
	**Reverse primer:** ATCTGTCCAGTAAGCTGGCTTCC	300 nM	
	**Probe:** FAM-CTCTACACTTGTAGTTCCG-BHQ-1	300 nM	
P. gingivalis 16S rRNA	**Forward primer:** CCGTAAGAATAAGCATCGGCTAACTC	300 nM	195 bp
	**Reverse primer:** CACGAATTCCGCCTGC	300 nM	
	**Probe:** FAM-CACTGAACTCAAGCCCGGCAGTTTCAA-BHQ-1	100 nM	

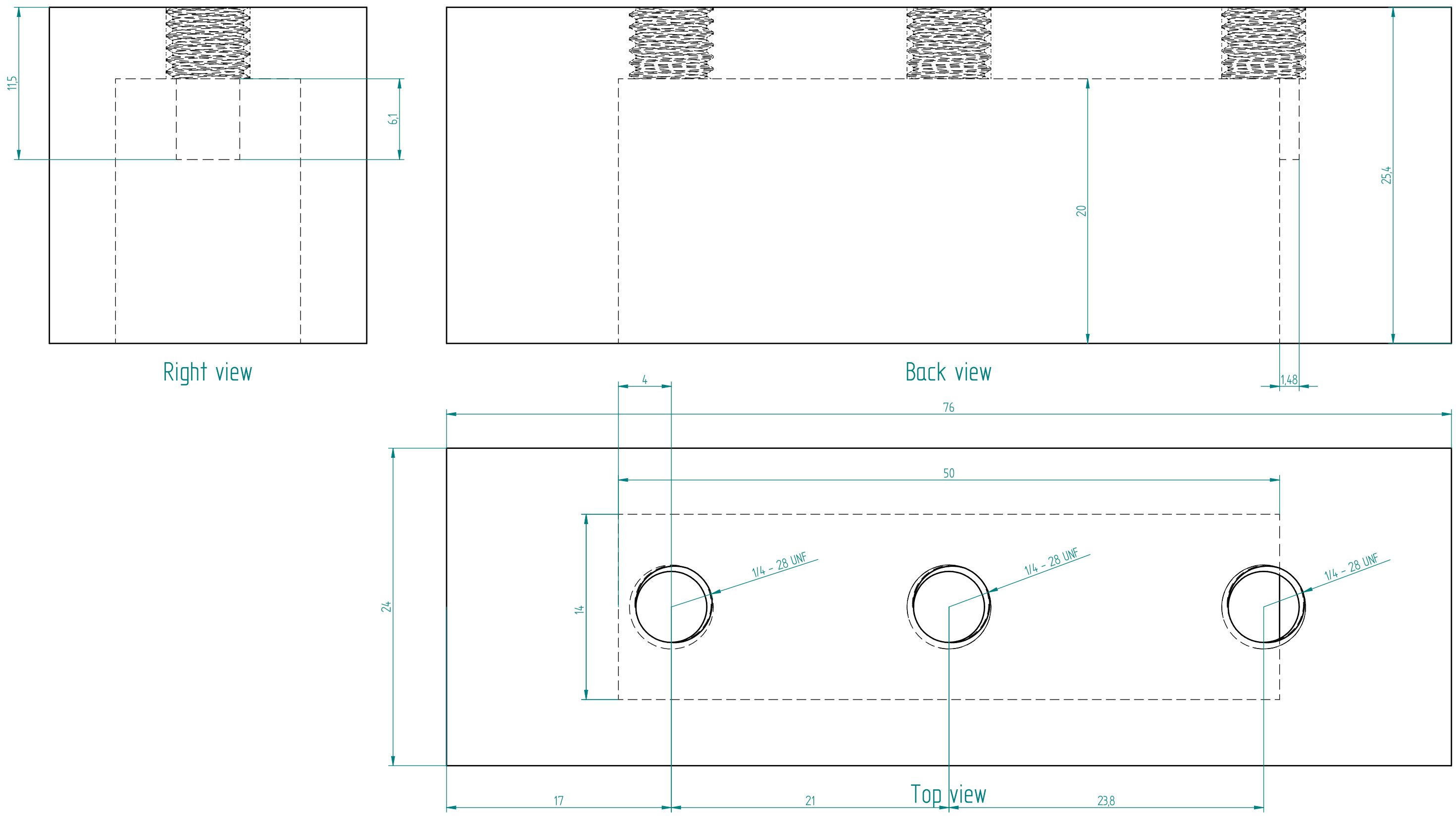

Figure A10: Dimensional drawings of the drip flow biofilm reactor.

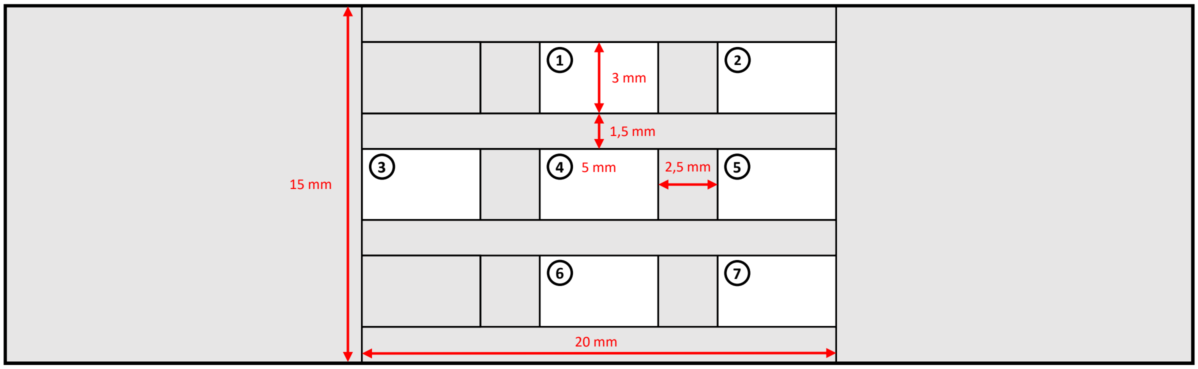

Figure A11: Locations on the biofilm substratum for confocal image analysis. In each of the seven white boxes, four different z-stacks are imaged.

References

(1) Vickerman MM, Mansfield JM, Zhu M, Walters KS, Banas JA. 2015. Codon-optimized fluorescent mTFP and mCherry for microscopic visualization and genetic counterselection of Streptococci and Enterococci. J Microbiol Methods 116:15–22.

(2) Shields RC, Kaspar JR, Lee K, Underhill SAM, Burne RA. 2019. Fluorescence tools adapted for real-time monitoring of the behaviors of *Streptococcus* species. Appl Environ Microbiol 85:e00620-19.

(3) Hansen MC, Palmer RJ, Udsen C, White DC, Molin S. 2001. Assessment of GFP fluorescence in cells of *Streptococcus gordonii* under conditions of low pH and low oxygen concentration. Microbiol 147:1383–1391.

(4) Shabayek S, Spellerberg B. 2017. Making Fluorescent Streptococci and Enterococci for Live Imaging. Methods Mol Biol 1535:141–159.

(5) Nicolle O, Rouillon A, Guyodo H, Tamanai-Shacoori A, Chandad F, Meuric V, Bonnaure-Mallet M. 2010. Development of SNAP-tag-mediated live cell labeling as an alternative to GFP in *Porphyromonas gingivalis*. FEMS Immunol Med Microbiol 59:357–363.

(6) Bachrach G, Haake SK, Glick A, Hazan R, Naor R, Andersen RN, Kolenbrander PE. 2004. Characterization of the Novel *Fusobacterium nucleatum* Plasmid pKH9 and Evidence of an Addiction System. Appl Environ Microbiol 70:6957–6962.

(7) Umana A, Nguyen TTD, Sanders BE, Williams KJ, Wozniak B, Slade DJ. 2022. Enhanced *Fusobacterium nucleatum* Genetics Using Host DNA Methyltransferases To Bypass Restriction-Modification Systems. J Bacteriol 204:1-18.

(8) Ponath F, Zhu Y, Cosi V, Vogel J. 2022. Expanding the genetic toolkit helps dissect a global stress response in the early-branching species *Fusobacterium nucleatum*. PNAS 119:1-12.

(9) Hartmann R, Jeckel H, Jelli E, Singh PK, Vaidya S, Bayer M, Rode DKH, Vidakovic L, Díaz-Pascual F, Fong JCN, Dragoš A, Lamprecht O, Thöming JG, Netter N, Häussler S, Nadell CD, Sourjik V, Kovács ÁT, Yildiz FH, Drescher K. 2021. Quantitative image analysis of microbial communities with BiofilmQ. Nat Microbiol 6:151–156.

(10) Verspecht T, Ghesquière J, Bernaerts K, Boon N, Teughels W. 2021. Evaluating the intrinsic

capacity of oral bacteria to produce hydrogen peroxide (H2O2) in liquid cultures: Interference by bacterial growth media. J Microbiol Methods 182:106170.

(11) Pesakhov S, Benisty R, Sikron N, Cohen Z, Gomelsky P, Khozin-Goldberg I, Dagan R, Porat N. 2007. Effect of hydrogen peroxide production and the Fenton reaction on membrane composition of *Streptococcus pneumoniae*. Biochim Biophys Acta Biomembr 1768:590–597.

(12) Takahashi N. 2015. Oral Microbiome Metabolism: From "Who Are They?" to "What Are They Doing?". J Dent Res 94:1628–1637.

(13) Herrero ER, Slomka V, Boon N, Bernaerts K, Hernandez-Sanabria E, Quirynen M, Teughels W. 2016. Dysbiosis by neutralizing commensal mediated inhibition of pathobionts. Sci Rep 6:38179.

(14) Kreth J, Zhang Y, Herzberg MC. 2008. Streptococcal Antagonism in Oral Biofilms: *Streptococcus sanguinis* and *Streptococcus gordonii* Interference with Streptococcus mutans. J Bacteriol 190:4632–40.

(15) Cheng X, Redanz S, Cullin N, Zhou X, Xu X, Joshi V, Koley D. 2018. Plasticity of the Pyruvate Node Modulates Hydrogen Peroxide Production and Acid Tolerance in Multiple Oral Streptococci. Appl Environ Microbiol, 84:1–15.
